# A functional screen with metformin identifies microRNAs that regulate metabolism in colorectal cancer cells

**DOI:** 10.1038/s41598-022-06587-9

**Published:** 2022-02-21

**Authors:** Ayla Orang, Saira R. Ali, Janni Petersen, Ross A. McKinnon, Amanda L. Aloia, Michael Z. Michael

**Affiliations:** 1 Flinders Health and Medical Research Institute – Cancer Program, Flinders University, Adelaide, South Australia 5042 Australia; 2 Cell Screen SA Facility, Flinders Health and Medical Research Institute, Flinders University, Bedford Park, South Australia 5042 Australia; 3 Department Gastroenterology and Hepatology, Flinders Centre for Innovation in Cancer, Flinders Medical Centre, Bedford Park, South Australia 5042 Australia

**Keywords:** Cancer metabolism, Gastrointestinal cancer, miRNAs, High-throughput screening

## Abstract

Metformin inhibits oxidative phosphorylation and can be used to dissect metabolic pathways in colorectal cancer (CRC) cells. CRC cell proliferation is inhibited by metformin in a dose dependent manner. MicroRNAs that regulate metabolism could be identified by their ability to alter the effect of metformin on CRC cell proliferation. An unbiased high throughput functional screen of a synthetic micoRNA (miRNA) library was used to identify miRNAs that impact the metformin response in CRC cells. Experimental validation of selected hits identified miRNAs that sensitize CRC cells to metformin through modulation of proliferation, apoptosis, cell-cycle and direct metabolic disruption. Among eight metformin sensitizing miRNAs identified by functional screening, miR-676-3p had both pro-apoptotic and cell cycle arrest activity in combination with metformin, whereas other miRNAs (miR-18b-5p, miR-145-3p miR-376b-5p, and miR-718) resulted primarily in cell cycle arrest when combined with metformin. Investigation of the combined effect of miRNAs and metformin on CRC cell metabolism showed that miR-18b-5p, miR-145-3p, miR-376b-5p, miR-676-3p and miR-718 affected glycolysis only, while miR-1181 only regulated CRC respiration. MicroRNAs can sensitize CRC cells to the anti-proliferative effects of metformin. Identifying relevant miRNA targets may enable the design of innovative therapeutic strategies.

## Introduction

Colorectal Cancer (CRC) is the third most common cancer worldwide and despite the progress in cancer screening and treatment, CRC still represents the second highest number of cancer-related deaths worldwide^1^. Despite the development of new targeted therapeutic options, survival outcomes for metastatic CRC remain modest, with lack of response or acquired resistance to therapy leading to treatment failure^2^.

Metformin is generally accepted as a respiration suppressor, mainly through targeting complex I within the mitochondrial electron transport chain and, thus, it leads to an imbalanced AMP/ATP ratio and metabolic stress^3^. Metformin activates AMPK and thus inhibits mTOR signalling, which ultimately leads to diminished protein synthesis and cellular proliferation. It also suppresses the mTOR pathway by hindering Akt activation, through AMPK-mediated phosphorylation of IRS-1^4^. Accumulating epidemiological evidence indicates that metformin plays a protective role for CRC incidence in diabetics and non-diabetic individuals^5,6^. Despite in vivo and in vitro studies confirming the anti-neoplastic benefits of metformin through direct targeting of cancer cells, uncertainty still exists regarding dose–response issues relevant to its clinical applications^7–9^. Furthermore, preclinical studies identifying specific mutations, such as in *STK11/LKB1*, that reduce cancer cell sensitivity to metformin, support the rationale for using drug combinations to enhance the response of cancer cells to prolonged treatment with metformin^10,11^. microRNAs (miRNAs) are post-transcriptional inhibitors of gene expression. They regulate a large variety of cellular processes and, therefore, their aberrant expression can contribute to human diseases, including cancer^12^. Several miRNAs were identified as tumour suppressors where a global decrease in miRNA expression correlated with increased grade of dysplasia^13^. Various studies show the potential of miRNAs as a class of cancer therapeutics^14^. Among several miRNA targets, metabolism-related mRNAs were shown to regulate different oncogenic pathways and represent new therapeutic targets^15^. Furthermore, altered metabolism profiles have been observed in the tumours of CRC patients^16,17^. miRNAs are able to rewire multiple metabolic pathways, simultaneously, by targeting several glycolytic and mitochondrial components and thereby regulate the initiation and progression of cancers (reviewed in^15^). However, in the case of CRC, the effect of miRNAs on glucose metabolism and oxidative phosphorylation needs further exploration. Furthermore, the effect of metabolism-regulating miRNAs on metformin sensitization in CRC is largely unknown. By inhibiting mitochondrial respiration, metformin provides an opportunity to dissect cellular metabolism and enable a functional genomics strategy, especially for investigating compensatory pathways, such as glycolysis. In this study, a functional synthetic miRNA mimic screen was performed to identify miRNAs with the ability of enhancing the anti-cancer properties of metformin, often by regulating energy production, impacting cell cycle progression and thereby slowing cell proliferation in CRC cells.

## Methods

### Cell lines

Colorectal cancer cell lines (HCT116, DLD1, RKO, HT29 and SW480) were obtained from American Type Culture Collection and cultivated in Dulbecco Modified Eagle Medium (DMEM) supplemented with 10% Fetal Bovine Serum (FBS). HKe-3 and HCT116 p53−/− cells were kindly donated by Professor Senji Shirasawa (Fukuoka University, Fukuoka, Japan) and Professor John Mariadason (Olivia Newton-John Cancer Research Institute, Melbourne, Australia).

### Functional miRNA screen

HCT116 cells were reverse-transfected, in 384-well plate format, with 25 nM final concentration of Dharmacon Human miRIDIAN miRNA Mimic Library 19.0 + 21.0 Supplement (Dharmacon). A siRNA targeting the PLK1 gene and a scrambled negative control mimic (Invitrogen) were used as positive or negative lethality controls, respectively**.** Transfection was performed with DharmaFECT 2 Transfection Reagent (Dharmacon) in Opti-MEM I Reduced Serum Media (Invitrogen). The screen was performed in duplicate: two replicates for miRNA mimic transfected and metformin treated plates (A, B) and two for miRNA mimic transfected and non-treated plates (C, D). 24 h post-transfection, 2.5 mM metformin treatment was added, followed by 72 h incubation. Proliferation assays, using both IncuCyte FLR (Essen) and xCelligence (ACEA Biosciences) real time cell analysis platforms, identified reproducible IC20 responses at this endpoint.

Treatment-induced changes in cell number and cell morphology were detected using the Operetta high-content imaging system (Perkin Elmer Life Sciences), along with Harmony software (Perkin Elmer Life Sciences), according to a method recently described by Massey^18^. Cells were labelled with 2 mM Hoechst 33342/well. Subsequently, cells were washed with 1 × PBS and then fixed by adding formaldehyde to a final concentration of 5% (v/v) in PBS and incubating for 20 min at room temperature.

The dynamic range was calculated between the positive and negative control using Z’ factor^19^. Raw values for each replicate were normalized to the average of the negative control raw values for the same plate, the normalized values for duplicates were averaged to get the final normalized value per treatment. To classify the hits, the fold change ratios of averaged normalized values, for samples relative to negative controls, were calculated. Hit identification was defined by calculating the percentage (ratio) of metformin treated cells versus untreated cells. Since metformin alone resulted in 20% reduction in cell viability, from the above calculation, those miRNAs showing a greater than 30% (i.e. further 10%) reduction in cell viability were selected as the preliminary hits. To stratify hits into priority outcome groups, according to set cut-offs for each bin, the following criteria were set: Among control/drug bins greater than 30%, the hits were selected if: (1) miRNAs show reasonable abundance; the average raw counts for the selected miRNA (unpublished small RNA-seq) were greater than 4 reads per million, either in metformin treated or untreated HCT116 cells, (2) miRNAs were from the same miRNA cluster or family, (3) miRNAs were reported to be up or downregulated by metformin treatment in the literature, or (4) miRNAs were involved in cancer-related pathways, biological processes, cellular compartments or molecular functions. miRNA Pathway Dictionary Database (miRPathDB) was used to perform the pathway and gene ontology analyses (https://mpd.bioinf.uni-sb.de/) and select those terms and pathways with strong evidence and FDR adjusted p value < 0.05^20^.

With selected miRNAs from the primary screen, a secondary screen was performed under the same conditions but with three replicates. To select hits from this validation screen data, the coefficient of drug interaction was calculated for each miRNA mimic as previously described^21^. The CDI values were classified as: (1) CDI ≤ 0.7 strong synergistic effect with metformin treatment, (2) 0.7 < CDI ≤ 1 synergistic effect with metformin, (3) 1 < CDI ≤ 1.1 additive effect with metformin, (4) CDI > 1.1 antagonistic effect with metformin^22^.

### Cell proliferation and cell apoptosis assay

Cells were reverse transfected in 96-well plates with miRNA mimics (GenePharama) using Lipofectamine 2000 (Invitrogen) and treated with 2.5 mM metformin for 3 days. Cell viability and apoptosis were assessed using crystal violet (Sigma) staining and Caspase Glo (Promega) assay, respectively. Proliferation validation experiments were performed using an xCELLigence Real Time Cell Analyzer platform (ACEA Biosciences). Cells were seeded in E-plates and cell proliferation was assayed as measures of cell index inferred by changes in impedence.

### Metabolic profiling

Extracellular Acidification Rate (ECAR) and Oxygen Consumption Rate (OCR) were measured with an XFe96 Extracellular Flux Analyzer (Seahorse Biosciences, Agilent) and used to monitor glycolysis and mitochondrial respiration rates, respectively. Briefly, HCT116 cells were reverse transfected with miRNA mimics in XFe96-well plates. One hour prior to assay, cells were equilibrated with bicarbonate-free, low buffered DMEM medium without any supplement, or supplemented with glucose or glutamine as indicated, in a 37 °C non-CO2 incubator. Mitochondrial respiration was analysed using Seahorse XF Mito Stress Test kits and glycolysis was measured using XF Glycolysis Stress Test kits all as per the manufacturer’s instructions (Agilent).

### Flow cytometry analysis of cell cycle

To define cell cycle distribution, flow cytometric analyses were performed. In brief, cells grown in 6 well plates were harvested by trypsinization and fixed with 80% ethanol. Cells were stained for total DNA content with a solution containing 50 µg/ml propidium iodide, 200 µg/ml RNase I and 0.1% Triton X-100 in PBS for 30 min. Cell cycle distribution was then assayed on a CytoFLEX flow cytometer (Beckman Coulter Life Sciences) and the data were analysed using FCS Express (De Novo software). A representation of the gating strategy is shown in Supplementary Fig. 8b.

## Results

### Identification of miRNAs that enhance the anti-proliferative effect of metformin

An unbiased high throughput functional screen was performed to identify miRNA mimics that can enhance the sensitivity of CRC cells to the anti-proliferative effect of metformin. HCT116 cells were treated with a dose of metformin (2.5 mM) equivalent to an IC20, without an enhanced effect on apoptosis (Supplementary Fig. 1). An IC20 dose was chosen to enable identification of miRNAs that can either enhance the effect of metformin or ameliorate the effect. At this sublethal dose, the effect of metformin on cell proliferation and cell apoptosis was not reliant on mutations in *TP53* or *KRAS* genes (Supplementary Fig. 1).

**Figure 1:**
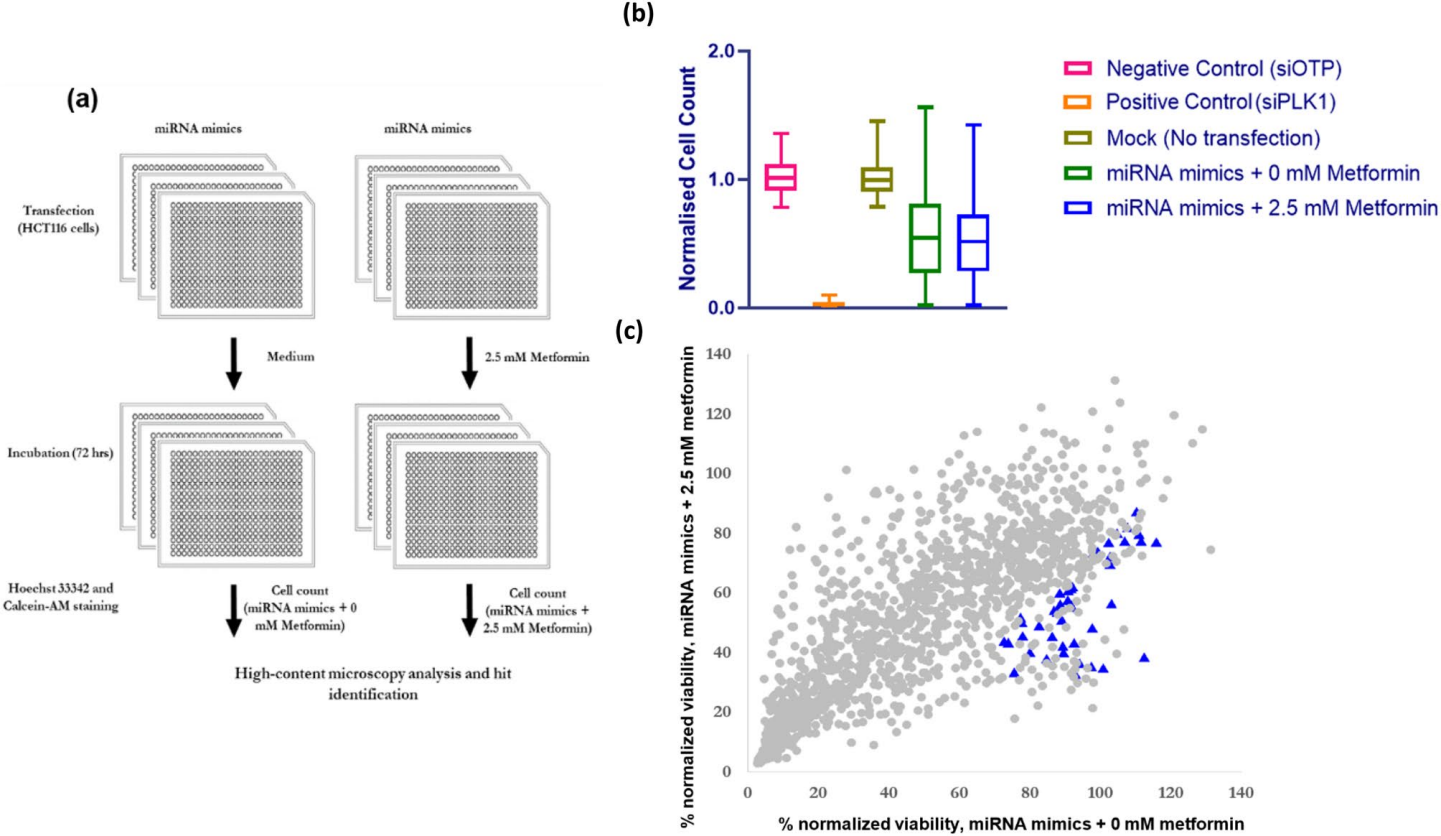
Schematic of high throughput screen of miRNA mimics, summary of data for CRC cell response to metformin treatment and scatter plot representation of the metformin sensitization screen with a miRNA mimic library. (**a**) Workflow of functional miRNA mimic screen is demonstrated. HCT116 cells were reverse transfected with 25 nM miRNA mimics and following 24 h incubation, cells were treated with 2.5 mM metformin or control medium for 72 h. Endpoint high-content image analysis was performed for cells stained with Hoechst 33342 and Calcein-AM. (**b**) Summary of viability data for the functional high throughput screen of miRNA mimics in combination with metformin is shown. Negative control for cell death is represented by the viability of cells transfected with negative control (OTP) and positive control is represented by the cells transfected with siRNA targeting PLK1. Mock control also shows the control for transfection (**c**) Percent viability of HCT116 cells reverse transfected with miRNA mimics and treated with 2.5 mM (y- axis) or 0 mM metformin (x-axis) are shown. The viability values were normalized against siOTP values and miRNAs selected for a secondary screen (blue triangles).

The screen replicates showed good reproducibility with Spearman *r* = 0.58 and 0.71 for metformin and vehicle treated groups, respectively (Supplementary Fig. 2). miRNA mimics were considered to act as a sensitizer if the viability of metformin treated cells transfected with mimics was ≤ 70% of the viability of the vehicle treated cells (without metformin) transfected with the non-targeting siRNA control (siOTP) (Fig. 1).

This threshold resulted in 153 metformin sensitising hits among miRNA mimics (Fig. 1c). To further refine the list and select the most robust hits, four additional rules were applied for selection, to enhance biological relevance of the miRNAs (as described in Materials and Methods). As a consequence, 49 miRNA mimics were selected (Fig. 1c, Supplementary File 2).

Selected miRNA mimics that enhanced the sensitivity of HCT116 cells to metformin, were further evaluated in a secondary high throughput screen, with similar workflow, in triplicate. Pairwise correlation of the replicates showed good reproducibility with Spearman *r* = 0.73, 0.76 and 0.74 for metformin treated and 0.73, 0.72 and 0.65 for vehicle treated groups (Supplementary Figs. 2 and 3).

Comparing the effect of combined treatment (miRNA mimic transfection and 2.5 mM metformin treatment) with the effect of metformin treatment alone or miRNA mimic transfection alone, 12 miRNAs were selected as they showed a strong synergistic effect with Coefficient of Drug Interaction < 0.7, *P* < 0.05 (Supplementary Fig. 4 and Table 1).

**Table 1:** Coefficient of drug interaction for secondary screen miRNAs.

miRNA	Viability: miRNA	Viability: miRNA + metformin	CDI
hsa-miR-145-3p	0.75	0.15	0.27
hsa-miR-676-3p	0.66	0.16	0.32
hsa-miR-1181	0.90	0.33	0.50
hsa-miR-376b-5p	0.54	0.20	0.51
hsa-miR-18b-5p	1.11	0.44	0.54
hsa-miR-3187-3p	0.57	0.25	0.59
hsa-miR-548v	0.41	0.19	0.64
hsa-miR-718	0.91	0.43	0.64
hsa-miR-3687	1.07	0.51	0.65
hsa-miR-99a-3p	0.92	0.46	0.67
hsa-miR-655-5p	0.56	0.28	0.68
hsa-miR-449c-5p	0.38	0.19	0.70

Values represent mean viability normalized to the siOTP control mean viability.

**Figure 2:**
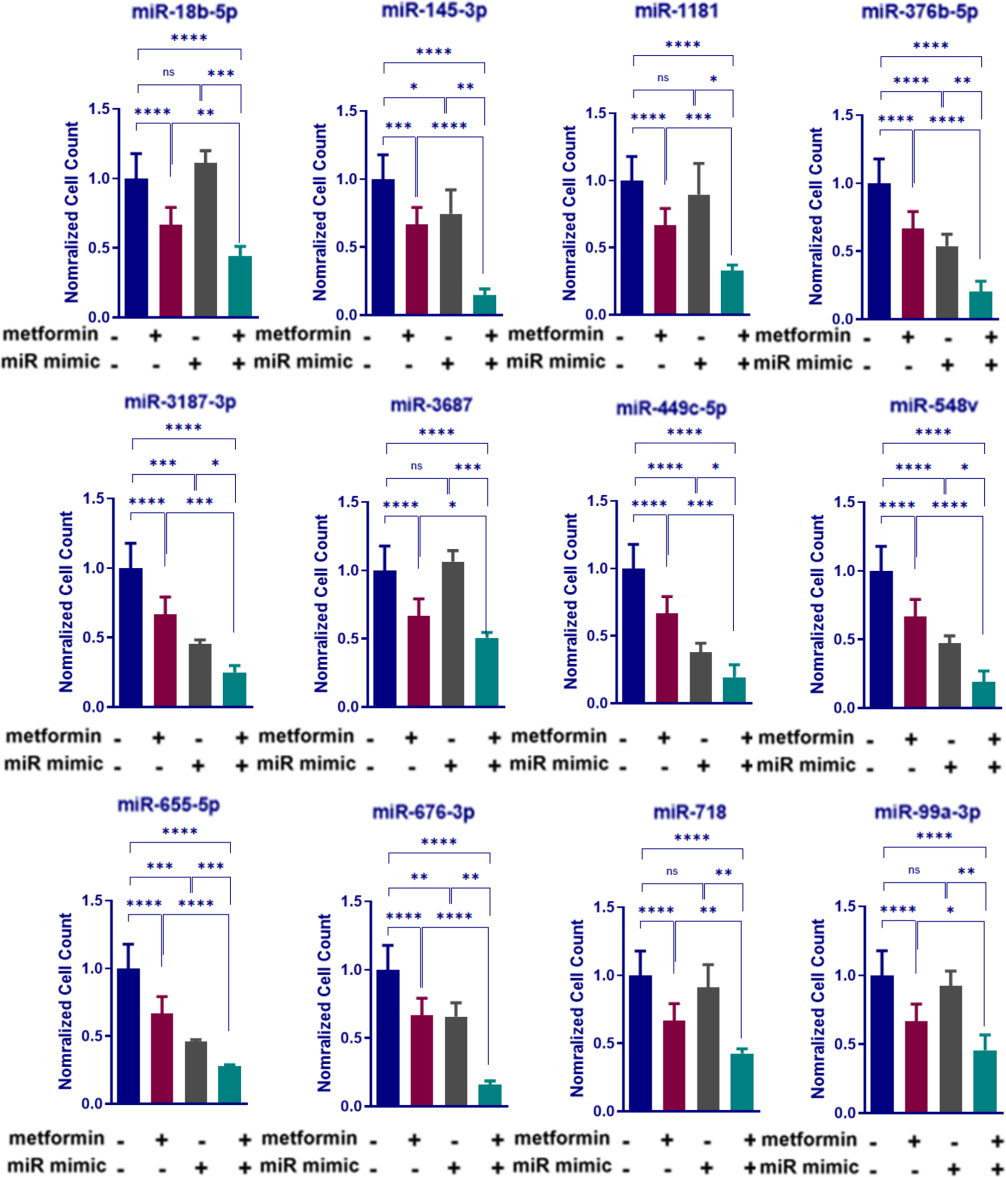
Viability of HCT116 cells transfected with miRNA mimics that show a strong synergetic effect with 2.5 mM metformin treatment. Normalized cell counts of HCT116 cells transfected with miRNA mimics, treated with 2.5 mM metformin and compared with 0 mM metformin treatment. Results are expressed as mean ± SD of 3 replicates and the statistical significance is indicated with asterisks (ns P > 0.05, *P ≤ 0.05, **P ≤ 0.01, ***P ≤ 0.001 and ****P ≤ 0.0001).

The proportional effects of microRNA activity and metformin, on cell proliferation, were further characterised to investigate synergistic responses. According to the normalized cell count, HCT116 cells treated with metformin and reverse transfected with negative control mimics showed about 30% reduction in viability compared with untransfected counterparts treated with medium (*P* < 0.0001, Fig. 2). While some miRNA mimic transfections in control medium resulted in little to no significant effect on viability of HCT116 cells, such as miR-1181, miR-18b-5p, miR-718, miR-3687 and miR-99a-3p, the combination of miRNA mimics and metformin treatment further reduced CRC cell viability, by approximately 55%, compared with cells transfected with NC mimics in control medium (*P* < 0.0001, Fig. 2). In summary, among other miRNAs that led to a reduction in cell viability in both 2.5 mM metformin treated groups and those in control medium, miR-145-3p and miR-449c-5p had the strongest synergistic effect in combination with metformin, reducing cell viability by 85% compared with HCT116 cells transfected with NC in control medium (*P* < 0.0001, Fig. 2).

### Real-time validation of metformin-sensitizing miRNAs: miRNA activity suppresses colorectal cancer cell proliferation

To further validate the secondary screen hits, HCT116 cells were transfected with the 12 selected miRNAs mimics (miR-145-3p, miR-18b-5p, miR-1181, miR-376b-5p, miR-3187-3p, miR-3687, miR-449c-5p, miR-548v, miR-655-5p, miR-676-3p, miR-718 and miR-99a-3p) or a negative control mimic and on the following day treated with 2.5 mM metformin or control medium for 3 days (Supplementary Fig. 5).

In HCT116 cells, transfection with 8 miRNA mimics enhanced growth inhibition by metformin as shown by a significant reduction in cell viability in cells transfected with mimics and treated with metformin, compared with cells treated with metformin alone or transfected with miRNA mimics alone (Supplementary Fig. 5a,c). These results were consistent with real-time cell index analysis as shown in Supplementary Fig. 5b,d. In summary, among the sensitizing miRNA mimics, transfected into HCT116 cells, miR-718, miR-145-3p and miR-376b-5p mimics had the most significant effect by reducing the cell index by 76, 70 and 69%, respectively, in the presence of 2.5 mM metformin while other miRNAs (miR-3187, miR-587v, miR-3687 and miR-449c-5p) didn’t show a synergistic effect in real-time cell index measurements (Supplementary Fig. 5b,d).

### Metformin sensitizing miRNAs can alter glycolysis

To investigate the role of the eight sensitizing miRNAs in regulating cancer glycolysis, a Seahorse XFe96 Extracellular Flux Analyzer quantified the generation of lactate, as monitored by the consequent extracellular acidification rate (ECAR). Furthermore, sequential compound injections such as glucose, oligomycin and 2-DG allowed calculation of glycolysis, glycolytic capacity and glycolytic reserve, respectively.

**Figure 3:**
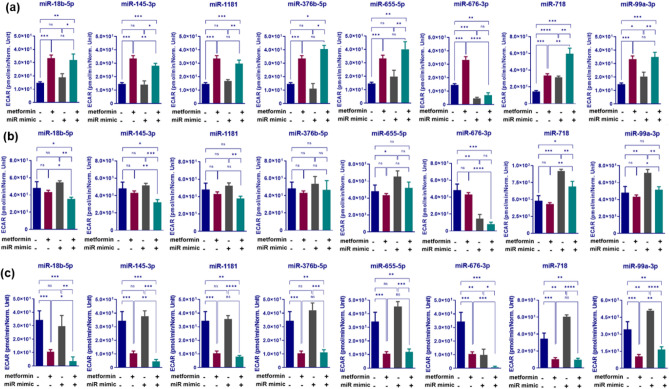
Changes in glycolysis (**a**), maximum glycolytic capacity (**b**) and glycolytic reserve (**c**) associated with miRNA mimic transfections in combination with metformin treatment of HCT116 cells. CRC cells were reverse transfected with selected miRNA mimics and treated with 2.5 mM metformin or control medium. Extracellular acidification rate (ECAR) of the cells was determined over a 30-min period using a Seahorse XFe-96 Instrument. The ECAR values were normalized to viability of the cells per group determined by crystal violet assay. Results are expressed as mean ± SD of 3 culture replicates and the statistical significance is indicated with asterisks (ns P > 0.05, *P ≤ 0.05, **P ≤ 0.01, ***P ≤ 0.001, ****P ≤ 0.0001.

2.5 mM metformin treatment of HCT116 cells resulted in a significant enhancement of glycolysis, as shown by 2.3 fold increase in HCT116 cells transfected with NC mimic and treated with 2.5 mM metformin for 3 days compared with NC mimic transfected cells in control medium (*P* = 0.002, Fig. 3a). Among the 8 miRNAs tested, transfection with miR-676-3p mimics resulted in a 69% decrease in glycolysis compared with NC mimic transfected HCT116 cells (*P* = 0.0001, Fig. 3a). The suppression effect was also significant enough to decrease glycolysis by 79% in samples that combined transfection of miR-676-3p mimics with metformin treatment, compared with those transfected with NC and treated with metformin (*P* = 0.0025, Fig. 3a). In addition, while transfection with miR-145-3p mimics alone had no significant effect on glycolysis compared with NC mimic transfection in control medium (*P* > 0.05), combination of miR-145-3p mimic transfection with metformin treatment, resulted in a similar effect (16% decrease) on glycolysis compared with metformin treated cells (*P* = 0.037, Fig. 3a). Although maximal glycolysis, resulting from ATP synthase inhibition, was not affected by metformin treatment of HCT116 cells compared with cells in control medium, combination of metformin treatment with miR-18b-5p or miR-145-3p mimic transfections introduced a significant decrease in glycolytic capacity by 18% or 25%, respectively, compared with metformin treatment alone (*P* = 0.011 and 0.004, respectively), Fig. 3b. Transfection with miR-676-3p in control medium showed a dramatic decrease in glycolytic capacity as compared with NC mimic transfection in control medium (70% increase, P = 0.0029) and, therefore, combination of miR-676-3p transfection with 2.5 mM metformin treatment represented a a synergistic effect by reducing maximal glycolysis compared with metformin treatment alone (80% decrease, P = 0.0002), Fig. 3b.

Metformin treatment resulted in a dramatic decrease in glycolytic reserve (3.3 fold, *P* = 0.0005), Fig. 3c. Investigating the effect of selected sensitizing miRNAs on glycolytic reserve revealed miR-676-3p to have inhibitory effect on glycolytic reserve compared with NC (by 71%, *P* = 0.0028) which led to a 92% reduction of glycolytic reserve in cells with metformin treatment compared with cells transfected with NC mimics and treated with metformin (*P* < 0.0001, Fig. 3c). Moreover, combination of miR-18b-5p or miR-145-3p mimic transfections with metformin treatment resulted in 66% and 57% decreases in glycolytic reserve compared with metformin treatment alone in HCT116 cells (*P* = 0.012 and 0.0089, respectively). This is in contrast to no significant effect on glycolytic reserve with miR-145-3p and miR-18b-5p mimic transfections in control medium (*P* > 0.05, Fig. 3c).

Altogether, these results showed transfection with miRNA mimics such as miR-676-3p, miR-145-3p or miR-18b-5p, in combination with metformin treatment in HCT116 cells, can diminish the glycolysis increase resulting from metformin treatment and further enhance the metformin mediated reduction of both glycolytic capacity and reserve.

### MicroRNAs can further modulate respiration in metformin-treated cells

The changes in oxidative phosphorylation of HCT116 cells associated with activity of the eight metformin sensitizing miRNAs, in combination with metformin treatment, were investigated. Seahorse XFe96 Extracellular Flux Analyzer quantified mitochondrial respiration in which key parameters of mitochondrial function such as basal and maximal respiration, respiratory reserve, ATP synthesis and coupling efficiency were measured by oxygen consumption rate (OCR) and calculated following three serial injections of compounds including oligomycin, FCCP, and rotenone/antimycin A.

**Figure 4:**
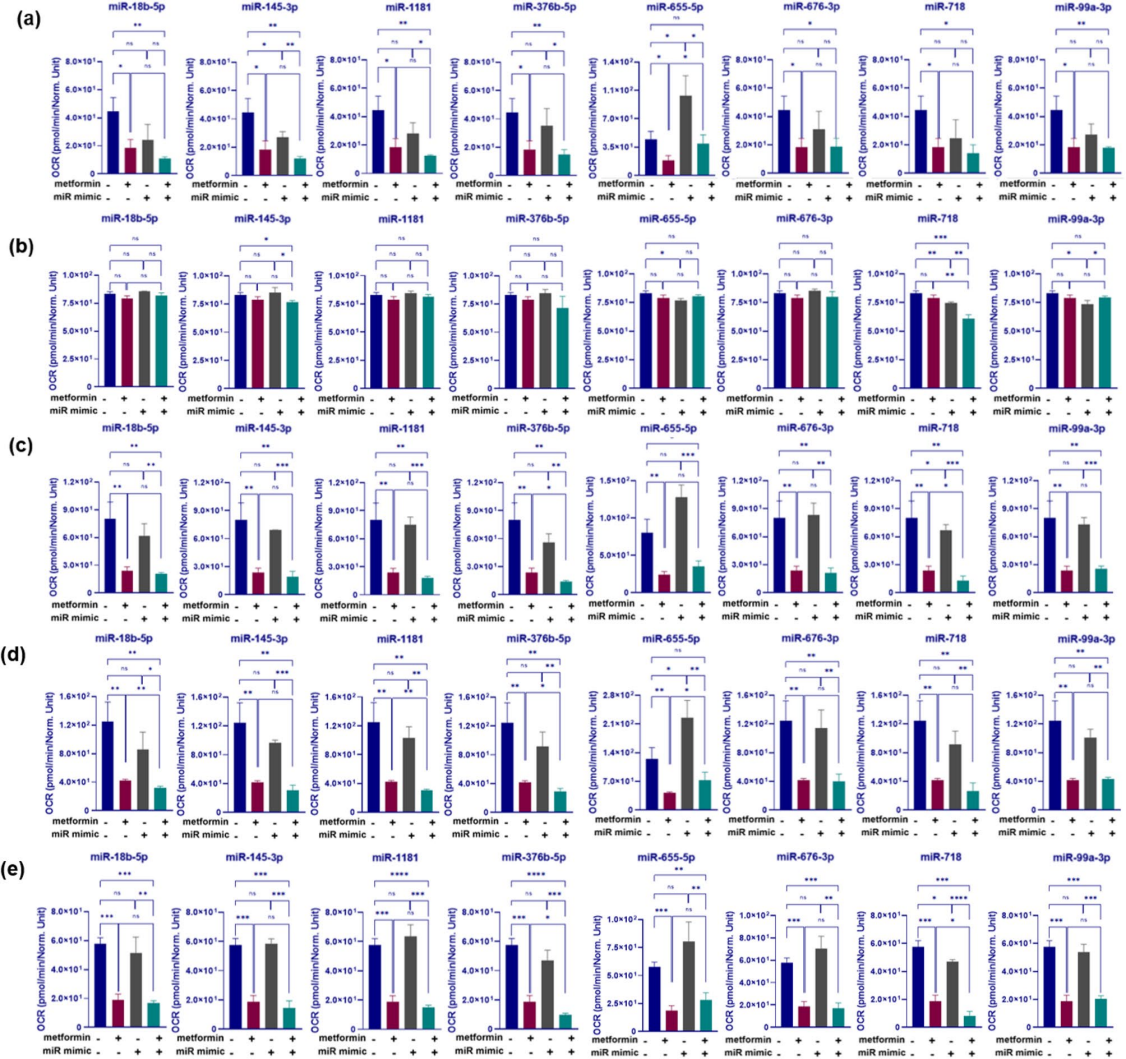
Changes in the reserved respiratory capacity (**a**), coupling efficiency (**b**), basal respiration (**c**), maximal respiratory capacity (**d**) and ATP synthesis (**e**) of HCT116 cells associated with miRNA mimic transfections in combination with metformin treatment. CRC cells were reverse transfected with selected miRNA mimics and treated with 2.5 mM metformin treatment or control medium. Oxygen consumption rate (OCR) of the cells was determined over a 30-min period using Seahorse XFe-96 instrument. The OCR values were normalized to viability of the corresponding cells acquired by crystal violet assay. Results are expressed as mean ± SD of 3 culture replicates and the statistical significance is indicated with asterisks (ns P > 0.05, *P ≤ 0.05, **P ≤ 0.01, ***P ≤ 0.001 and ****P ≤ 0.0001).

Since metformin treatment alone resulted in a dramatic fall in almost all mitochondrial function including basal respiration, maximal respiration, respiratory reserve and ATP synthesis (by 71%, 66%, 59% and 68% with *P* = 0.0064, 0.0065, 0.017 and 0.0004, respectively, Fig. 4) compared with cells in control medium, combining miRNA transfections with metformin treatment had little or no further enhancement effect on some of these parameters. For instance, there was no enhancement in reserved capacity with all 8 mimic transfections investigated in combination with metformin treatments (*P* > 0 0.05, Fig. 4a). Notably, miR-655-5p transfection, alone, generated a significant induction in OCR (1.2- fold, P = 0.02), and managed to negate the OCR reduction seen with metformin alone (Fig. 4a). Furthermore, only miR-718 mimic transfections led to a 10% reduction in coupling efficiency compared with NC mimic transfections (*P* = 0.0025). When these transfections were combined with metformin treatment there were further 17% and 12% reductions in coupling efficiency compared with NC mimic transfections in control medium or medium with 2.5 mM metformin, respectively (*P* = 0.0007, 0.002, respectively), Fig. 4b.

With regard to basal respiration, transfection of miR-376b-5p or miR-718 in combination with metformin treatment resulted in a 82 or 84% reduction in basal respiration compared with NC transfected cells in control medium (*P* = 0.0033 and 0.0035, respectively), Fig. 4c. The effect was also significant in comparison with metformin treatment alone as shown by 41 and 46% reduction in basal respiration in cells transfected with miR-376b-5p and miR-718 and treated with metformin (*P* = 0.02 and 0.05, respectively), Fig. 4c.

To investigate the effect of miRNAs on maximal respiration, where oxidative phosphorylation operates at maximum capacity, ATP synthesis was disrupted by an uncoupler treatment (FCCP). While metformin resulted in a significant reduction in maximal respiration (by 66%, *P* = 0.0065), combination of miR-18b-5p, miR-1181 and miR-376b-5p transfection with metformin treatment led to a further 8.8, 9.3 and 11% decrease in the maximal respiratory capacity of HCT116 cells, respectively, compared with metformin treatment alone (*P* = 0.005, 0.001 and 0.01 respectively), Fig. 4d. Notably, miR-655-5p transfection alone significantly increased maximal respiration capacity, however this was also inhibited by metformin (P = 0.02, P = 0.04, respectively), Fig. 4d.

Both miR-376b-5p and miR-718 had similar effects on ATP synthesis, decreasing ATP turnover by 47 and 58% in combination with metformin and compared with HCT116 cells transfected with NC mimics and treated with metformin (*P* = 0.025 and 0.028, respectively), Fig. 4e. In summary, these results indicate that miRNAs such as miR-376b-5p, miR-718, miR-18b and miR-1181 contribute to mitochondrial function by regulating different respiration parameters such as basal and maximal respiration as well as ATP turnover.

**Figure 5:**
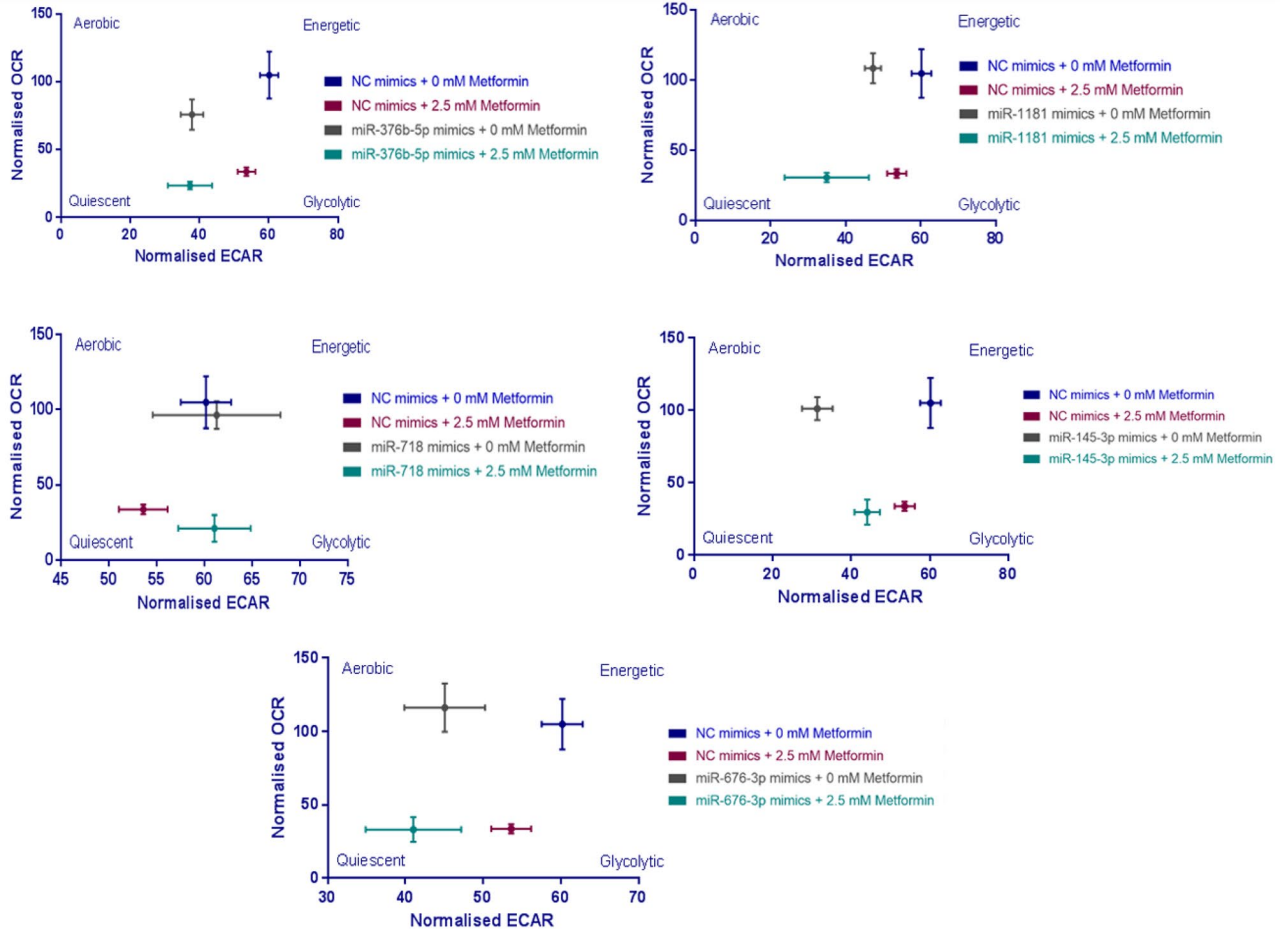
Integrated energy maps of HCT116 cells associated with miRNA mimic transfections in combination with metformin treatment. CRC cells were reverse transfected with selected miRNA mimics and treated with 2.5 mM metformin treatment or control medium. The baseline oxygen consumption rate (OCR) and extracellular acidification rate (ECAR) calculated were combined to generate the basal phenogram. The OCR and ECAR values were normalized to viability of the corresponding cells acquired by crystal violet assay. Results are expressed as mean ± SD of 3 technical replicates.

By combining the baseline oxygen consumption rate (OCR) and extracellular acidification rate (ECAR) measurements across experiments, an integrated bioenergetic phenogram could be constructed for each combination of miRNA mimic and metformin treatment (Fig. 5). The phenograms highlighted the combinatorial effect of metformin with miR-1181 and, especially, miR-676-3p in depleting energy production in HCT116 cells and forcing them into quiescence (Fig. 5).

### Metformin sensitizing miRNAs influence apoptosis and cell cycle

To investigate consistency in the roles of six out of the eight metformin sensitizing miRNAs that regulate colorectal cancer cell metabolism, miRNA activity was increased by transfecting a panel of CRC cell lines (RKO, DLD1, HT29 and SW480), with miRNA mimics following 2.5 mM metformin treatment and cell viability was quantified. Among other CRC cell lines tested, RKO, DLD1 and HT29 cell transfections with miR-18b-5p, miR-145-3p, miR-1181, miR-376b-5p, miR-676-3p and miR-718 mimics resulted in enhanced sensitivity of cells to the anti-proliferative effect of metformin (Supplementary Fig. 6). However, the SW480 cell line showed little sensitivity to several of these miRNAs. Only transfection with miR-18b-5p or miR-676-3p increased sensitivity of SW480 to metformin by decreasing the viability of the cells by 26% or 43% in combination with metformin (*P* = 0.01 and 0.004, respectively), Supplementary Fig. 6.

To investigate the roles of miR-1181, miR-18b-5p, miR-145-3p, miR-376b-5p, miR-676-3p and miR-718 mimics in combination with metformin on CRC cell death, caspase 3/7 activity was assessed as a marker of apoptosis. While transfection of four responsive CRC cell lines with miR-18b-3p, miR-145-3p, miR-1181, miR-376b-3p and miR-718 mimics, in combination with metformin treatment, showed no consistent pro-apoptotic function in CRC cell lines, miR-676-3p did exert a pro-apoptotic function by increasing caspase 3/7 activation by 103%, 36% and 47% in control medium and 103%, 58% and 71% in combination with metformin in HCT116, DLD1 and HT29 cell lines, respectively (P < 0.05 HCT116, P < 0.05 for DLD1 and P < 0.001 for HT29, respectively), Supplementary Fig. 7. Among four cell lines tested for the combinatorial effect of metformin treatment and miRNA mimic transfections on programmed cell death, RKO showed the least sensitivity with no significant increase in caspase3/7 activity following the combination of metformin treatment and miRNA mimic transfections, while HT29 cells had the highest sensitivity where a combination of 2.5 mM metformin treatment and miR-18b-5p, miR-1181, miR-376b-5p, miR-676-3p or miR-718 mimics resulted in 46, 87, 42, 123 and 60% increases in caspase 3/7 activity compared with NC mimics in control medium (P = 0.0003, < 0.0001, = 0.0006, < 0.0001 and = 0.0008, respectively), Supplementary Fig. 7. Taken together, these results indicate that except for miR-676-3p, which sensitizes CRC cells to the growth-inhibiting properties of metformin, most likely through increased apoptosis, other miRNAs induce the anti-proliferative effect of metformin but do not enhance caspase-dependent apoptosis in the presence of metformin.

**Figure 6:**
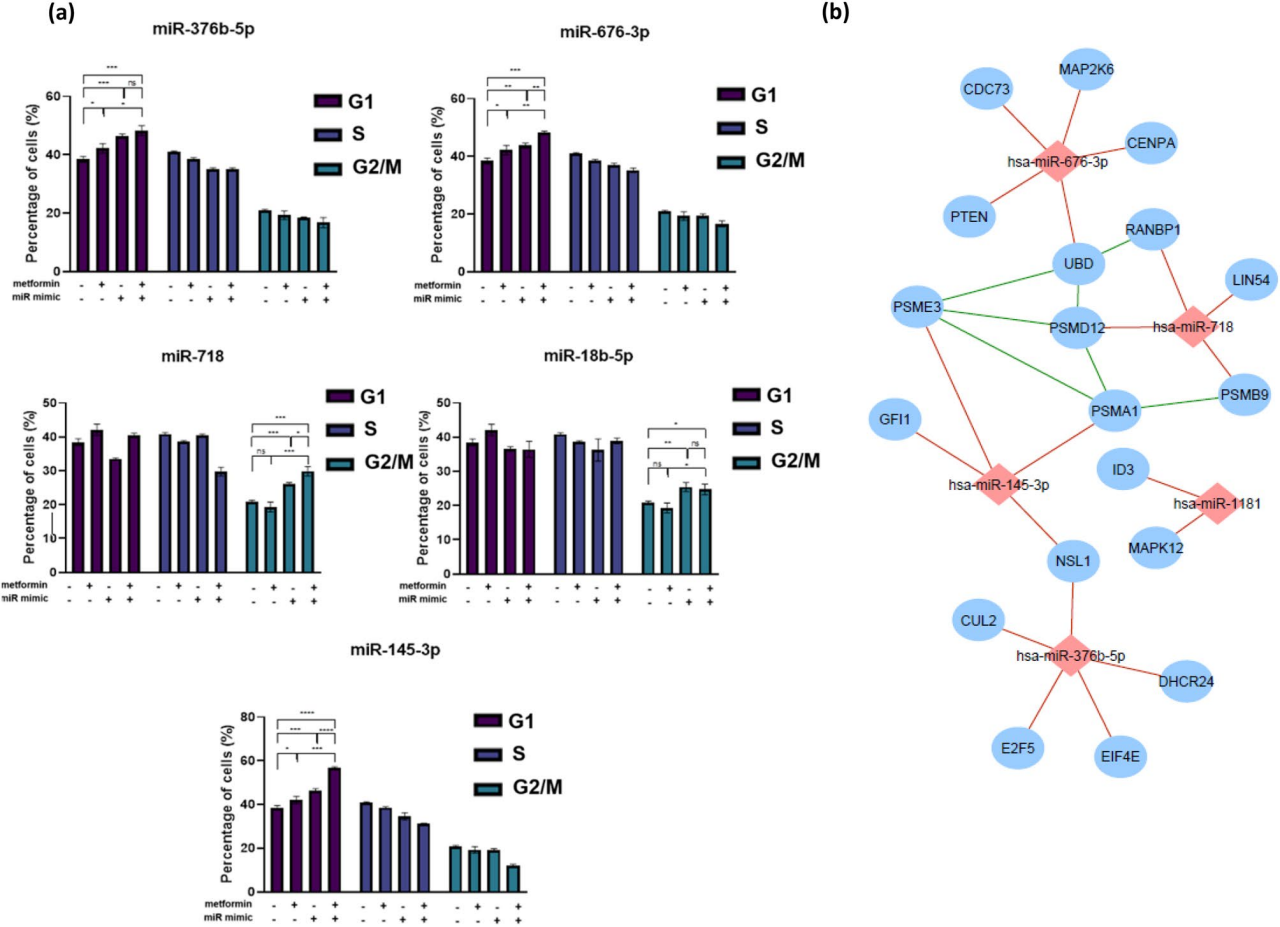
Cell cycle distribution of HCT116 cells after transfection with sensitizing miRNAs and treatment with metformin or control medium for 96 h (**a**) and the miRNA-based sub-network of putative target genes associated with cell cycle (**b**). (**a**) Histograms showing the percentage of HCT116 cells transfected with miR-18b-5p, miR-145-3p, miR-376b-5p, miR-676-3p and miR-718 mimics and treated with 2.5 mM metformin for 72 h in G0/G1, S, and G2/M phases, compared with cells in control medium. A “–” miRNA mimic status indicates transfection with NC negative control mimics. Results are expressed as mean ± SD of 3 replicates and the statistical significance is indicated with asterisks (ns P > 0.05, *P ≤ 0.05, **P ≤ 0.01, ***P ≤ 0.001, ****P ≤ 0.0001). (**b**) This is an organic layout of the integrated network associated putative genes organised in cell cycle associated ontologies acquired by Targetscan predition algorithm and NetworkAnalyst-Panther/Slim GO analysis. The miRNA-gene interactions are shown as red lines while gene–gene interactions are green. The blue and red nodes represent predicted taget genes and miRNAs, respectively.

To examine whether the growth inhibitory effect of the six miRNAs (miR-145-3p, miR-18b-5p, miR-1181, miR-376b-5p, miR-676-3p and miR-718) on HCT116 cells was partly due to cell cycle changes, cell cycle analysis was performed. Notably, 2.5 mM metformin treatment resulted in partial G1 cell cycle arrest by inducing the percentage of cells in G1 phase by 4% compared to vehicle treated cells (*P* = 0.03, Fig. 6a).

Transfection with miR-376b-5p, miR-676-3p and miR-145-3p mimics alone enhanced the percentage of cells at G1 phase compared to vehicle treated cells (8, 5 and 8%, *P* = 0.0006, 0.0026 and 0.0006, respectively), Fig. 6a. Combination of miR-676-3p and miR-145-3p mimics with 2.5 mM metformin resulted in 10% and 18% increase in G1 cells, respectively, which was significantly higher compared to metformin treatment or miRNA mimic transfection alone (Fig. 6a and Supplementary Fig. 8, *P* < 0.01).

While 2.5 mM metformin had no significant effect on the proportion of cells in G2/M phase, miR-18b-5p and miR-718 mimic transfections alone resulted in an increase in the percentage of cells in G2/M phase by 5% (*P* = 0.001 and 0.0004, respectively), Fig. 6a. The combination of metformin treatment with miR-718 mimic transfection resulted in a 4% further increase in G2/M phase cells compared to miRNA mimic treatment alone (*P* = 0.01). These data suggest the ability of metformin sensitizing miRNAs, such as miR-676-3p and miR-145-3p, in cooperating with metformin to delay cell cycle progression, mainly at G1 phase. Moreover, some miRNAs such as miR-18b-5p and miR-718 exert their metformin-sensitizing effect partially through blocking cells in the G2/M phase (Fig. 6a and Supplementary Fig. 8).

To identify likely pathways that are affected by the metformin sensitizing miRNAs, gene ontology analyses of putative targets revealed cell cycle to be a common process regulated by these miRNAs, with genes such as those involved in proteasome activity (*PSMB9*, *PSMA1*, *PSME3*, *PSMD12* and *UBD*) as well as some well known cell cycle regulating genes such as genes in the MAPK signalling pathway and cell division (Fig. 6b).

## Discussion

Metformin treatment leads to a self-limiting accumulation in cancer cells, theoretically, through inhibiting proton pumping, depolarizing the mitochondrial membrane and, therefore, limiting the accumulation of the drug^23^. Metformin inhibits mTORC1 through both an AMPK- dependent and independent manner^4^. Also, chronic inhibition of mTORC1 was shown to relieve the negative feedback loop from the S6K-IRS1 axis leading to hyperactivation of mTORC2 and, consequently, Akt activation. Therefore, inhibition of mTORC1, located upstream of S6K in the pathway, prevents the inhibitory feedback on IRS-1 and leads to an increased IGF-1R-mediated proliferation signal^24^. Altogether, these results emphasize the potential for a combination therapy that can increase the sensitivity of cancer cells to metformin. Given the involvement of miRNAs in key cancer hallmarks, including aberrant cellular metabolism, the current study investigated the ability of miRNAs to sensitize colorectal cancer cells to the anti-proliferative effect of metformin. Of the 2063 miRNAs that were tested through primary and secondary high throughput functional screens, the strong synergistic effects of 8 miRNAs, with metformin, were validated in several CRC cell lines (CDI < 0.7). Among 8 validated hits, transfection of miR-145-3p, miR-676-3p, miR-655-5p, miR-18b-5p and miR-376b-5p into CRC cells not only repressed tumour cell proliferation, but also potentiated the efficacy of metformin in repressing cancer cell division.

While this study concentrated on miRNAs that enhance the metformin effect, those that suppress metformin responses may reveal more about metformin’s mechanism of action. In vivo investigation of these metformin sensitizing miRNAs may identify systemic roles and provide pre-clinical data for novel therapeutic strategies.

To test the roles of the identified sensitizing miRNAs through mechanisms related to cellular metabolism, including cancer cell glycolysis and mitochondrial respiration, the changes in metabolic profile associated with metformin treatment and altered miRNA activity were investigated. Six out of eight metformin sensitizing miRNAs (miR-376b-5p, miR-1181, miR-18b-5p, miR-676-3p, miR-718 and miR-145-3p) were identified as regulators of glycolysis or mitochondrial respiration.

Consistent with previous studies, metformin-mediated inhibition of respiration resulted in a compensatory increase in glycolysis but also a dose dependent decrease in glycolytic capacity and reserve^25^. This may indicate the pleiotropic nature of metformin and suggests direct targeting of molecules involved in regulation of CRC cell glycolysis. In this study, investigating different parameters of glycolysis revealed miR-676-3p and miR-376b-5p are suppressors of glycolysis, glycolytic capacity and glycolytic reserve and showed the ability of these miRNAs to hinder the metformin-mediated increase in glycolysis. Moreover, the addition of oligomycin, which blocks mitochondrial ATP production, showed that increased miR-676-3p activity in CRC cells enhanced the inhibitory effect of metformin on glycolytic capacity and reserve. While some studies showed the diagnostic value of miR-676-3p in cancers such as gastric, prostate and breast cancer^26–28^, very little is known about the function of this miRNA. Li et al., demonstrated the inhibitory effect of miR-376b-5p on angiogenesis in middle cerebral artery occlusion by targeting the HIF-1α-mediated VEGFA signalling pathway^29^. Low expression of miR-376b-5p was also shown to be correlated with poor prognosis of patients with pancreatic adenocarcinoma^30^.

Moreover, miRNAs such as miR-18b-5p and miR-145-3p were demonstrated to have a suppressor effect on glycolytic properties such as glycolytic rate, glycolytic capacity and glycolytic reserve in combination with metformin whilst exerting no significant effect alone. miR-18b-5p was shown to be differentially expressed in certain cancers^31–33^. However, there are discrepancies with regard to the functions of this miRNA in cancer, as it can be both a tumour suppressor and oncogenic miRNA. In melanoma cells, this miRNA was downregulated and was shown to directly target the proto-oncogene MDM2 and regulate DNA methylation and, thereby, regulate the p53 signalling pathway to reduce tumour cell growth and induce cell death^34^. However, the oncogenic activity of miR-18b-5p was shown in breast cancer cells where miR-18b-5p targets DOCK4, a significant cell division factor mainly involved in the regulation cell adhesion^35^. miR-145-3p, generally considered the passenger strand of mature miR-145, was shown to be downregulated in different cancer types and is also reportedly a tumour suppressor and regulator of tumour growth, cell death and tumour metastasis^36–39^. Caution should be exercised in over-interpreting the biological relevance of pre-miR-145-derived miRNAs, as their expression in epithelial cells is contentious^40^ and found to be low in this study, however this doesn’t limit their therapeutic potential nor their ability to identify other drug targets. Even in the absence of metformin, transfection of miRNAs such as miR-718 and miR-99a-3p enhanced glycolysis, while miR-676-3p suppressed the glycolytic properties of CRC cells.

As a direct target of metformin, mitochondrial function has been shown to be affected by metformin treatment in different cancers^23,41,42^. Accordingly, 2.5 mM metformin treatment of CRC cells resulted in a dramatic decrease in mitochondrial parameters, such as basal and maximal respiration.

The maximum capacity of HCT116 cell respiration, measured from the baseline in the presence of oligomycin and then FCCP, was lower in cells transfected with miR-18b-5p, miR-1181 or miR-376b-5p and treated with metformin, compared with NC mimic transfected plus metformin treated cells. miR-376b-5p and miR-718 showed the strongest effect on mitochondrial parameters by further enhancing the inhibitory effect of metformin on basal respiration and ATP turnover. Hypoxia-inducible miR-1181 is a known tumour suppressor in cancers such as pancreatic, ovarian and prostate cancer and is shown to target *SOX2*, *STAT3* and *HOXA10* to inhibit proliferation, migration and invasion and to promote EMT^43–47^. miR-718 was originally identified in 2006 and Leng et al., showed the anti-cancer role of this miRNA, by targeting *VEGF*, in ovarian cancer^48,49^. The tumour suppressive function of miR-718 was then confirmed in cancers including hepatocellular carcinoma and oesophageal squamous cell carcinoma, as well as thyroid and ovarian cancers; it was shown to inhibit cancer cell proliferation, invasion and to regulate innate immune response by targeting *VEGF*, *PDPK1*, *IRAK1*, *EGR3* and *PTEN*^49–53^. Similar to the findings in this study, miR-718 supressed cancer cell metabolism and energy production in papillary thyroid cancer through regulating the PI3K/Akt/mTOR pathways^54^.

Investigation of the anti-tumour properties of metformin, in combination with miRNAs that regulate CRC cell metabolism, revealed 6 metabolism-regulating miRNAs that enhanced the anti-proliferative effect of metformin. However, the effect of these miRNA mimics on CRC cell viability is largely due to cellular processes other than apoptosis, as only miR-676-3p showed pro-apoptotic activity in HCT116, DLD1 and HT29 cells.

Cell cycle arrest is often a major indicator of anti-cancer drug activity^55^. These results showed that metformin suppresses cell proliferation by increasing the proportion of CRC cells in G1 phase. They are consistent with several previous studies showing that metformin induces cell cycle arrest without affecting cell apoptosis in different cancers^56–59^. Furthermore, cell cycle distribution was adversely affected by some of the metformin sensitizing miRNAs such as miR-376b-5p, miR-18b-5p, miR-676-3p, miR-145-3p and miR-718. Investigation of the combinatorial effect of metformin-sensitizing miRNAs on cell cycle regulation revealed some miRNAs, notably miR-676-3p and miR-145-3p, cooperate with metformin to induce further cell cycle arrest at G1 phase. The combination of aforementioned miRNAs with metformin treatment prevented HCT116 cells from entering S phase as the percentage of cells in S phase was lower compared with the control medium, metformin treatment alone or miRNA mimic transfection alone. This is likely to reflect arrested mitosis and suppressed cell proliferation^60^. Similarly, Goto et al., showed miR-145-3p, as the passenger strand, is downregulated in cisplatin-resistant prostate cancer and is involved in cell cycle regulation through targeting cell cycle regulators such as CDK1^61^.

Although metformin treatment did not affect cells in G2/M phase, miR-18b-5p and miR-718 both resulted in cell cycle delay in G2/M phase when combined with metformin. Consistent with our findings, *CCNB1* (G2/M specific cyclin B1) has been validated as a target of miR-718, which supports the ability of miR-718 to regulate cell cycle progression and, thus, cell proliferation^62^. Identifying the miRNA targets that are responsible for these observations may highlight useful adjuvant drug targets for slowing tumour growth. Bioinformatic predictions suggest several candidate targets, some with major roles in metabolic pathways. Experimental validation of these targets is required to generate novel combination therapies that may unlock the anti-cancer potential of metformin.

## Conclusions

By using metformin to suppress oxidative phosphorylation, and thereby force reliance on glycolysis, this study has identified miRNAs that regulate basic metabolic processes in mammalian cells. As metformin, itself, can slow the proliferation of CRC cells, this study was designed to identify specific miRNAs that further enhance the anti-proliferative properties of metformin with therapeutic intent. It has uncovered an additional layer of miRNA regulation, whereby metformin-sensitizing miRNAs suppress CRC cell metabolism through the regulation of glycolysis or respiration, sometimes solely in the presence of metformin. Further investigation is required to elucidate the precise molecular mechanisms by which these miRNAs target metabolism-associated genes.

## Supplementary Information

Supplementary Figures.

Supplementary Information 1.

## Data Availability

The dataset supporting the conclusions of this article is included within the article and its additional files.

## Abbreviations

AMP: Adenosine monophosphate
AMPK: AMP-activated protein kinase
ATP: Adenosine triphosphate
CCNB1: Cyclin B1
CDI: Coefficient of drug interaction
CRC: Colorectal Cancer
DOCK4: Dedicator of cytokinesis 4
ECAR: Extracellular acidification rate
EGR3: Early Growth Response 3
EMT: Epithelial-to-mesenchymal transition
FCCP: Carbonyl cyanide-p-trifluoromethoxyphenylhydrazone
HIF-1: Hypoxia-inducible factor 1
HOXA16: Homeobox Protein Hox-1B
IGF-1R: Insulin-like growth factor I receptor
IRAK1: Interleukin 1 Receptor Associated Kinase 1
IRS-1: Insulin receptor substrate 1
miRNA: MicroRNA
mTORC: Mechanistic target of rapamycin complex
NC: Negative control
OCR: Oxygen consumption rate
PDPK1: Phosphoinositide-dependent kinase 1
PSMA1: Proteasome 20S subunit alpha 1
PSMB9: Proteasome 20S subunit beta 9
PSMD12: Proteasome 26S subunit, non-ATPase 12
PSME3: Proteasome activator subunit 3
PTEN: Phosphatase and tensin homolog
S6K: Ribosomal protein S6 kinase
SOX2: SRY-box transcription factor 2
STAT3: Signal transducer and activator of transcription 3
STK11/LKB1: Serine/threonine kinase 11
UBD: Ubiquitin D
VEGFA: Vascular endothelial growth factor A
