# Supplementary material for: A functional screen with metformin identifies microRNAs that regulate metabolism in colorectal cancer cells
Source: Sci Rep. 2022 Feb 21;12:2889. doi: 10.1038/s41598-022-06587-9 (PMC8861101; doi:10.1038/s41598-022-06587-9)
Supplement: Supplementary file 1 — Supplementary Figures. [file 41598_2022_6587_MOESM1_ESM.docx]

# Supplementary Figures


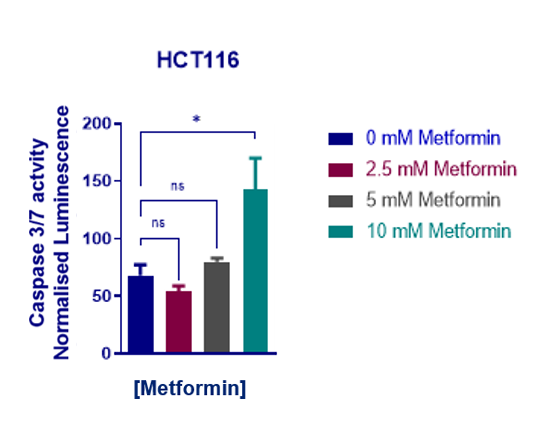

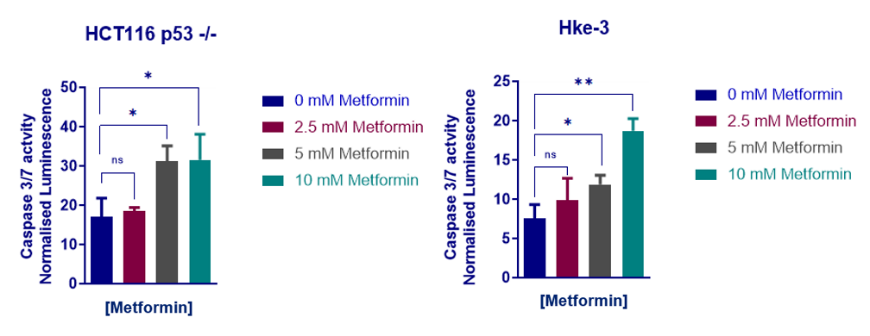

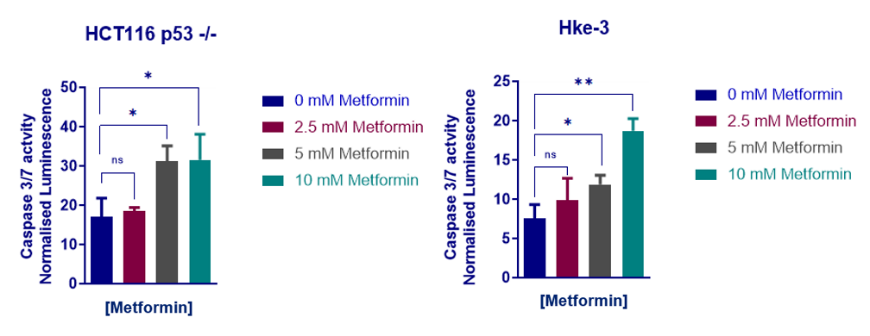

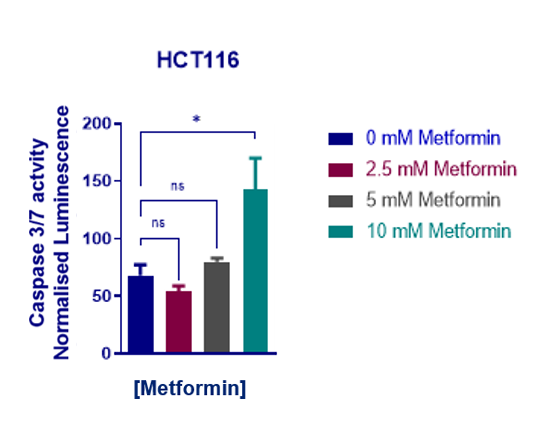

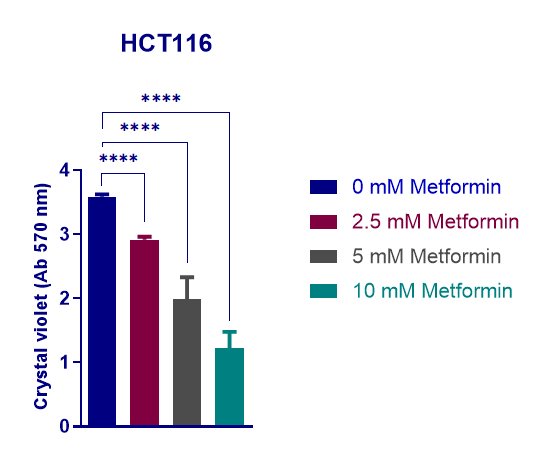

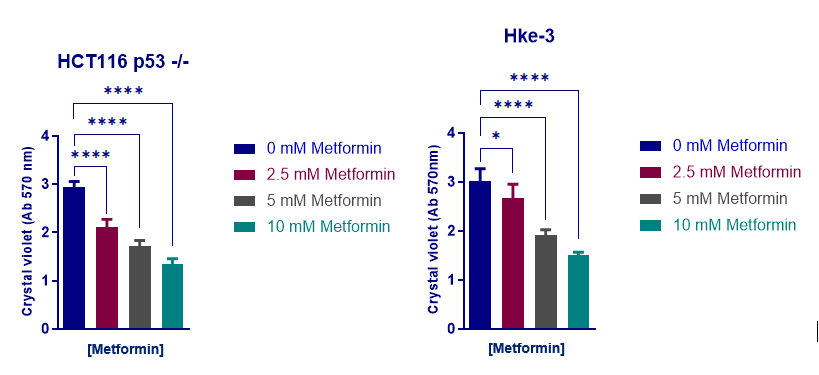

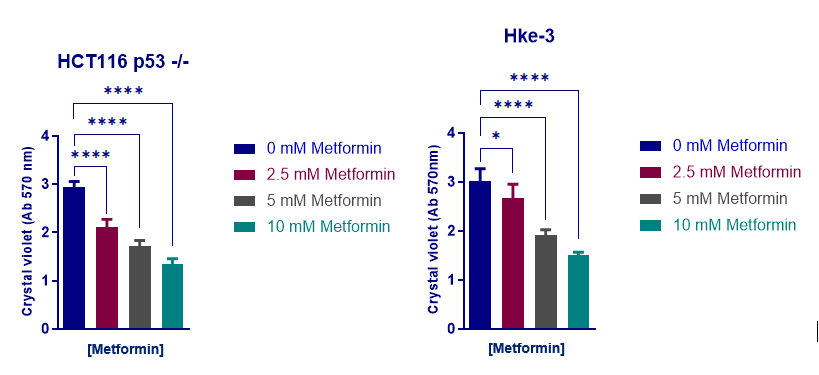


**(a)**

**(b)**


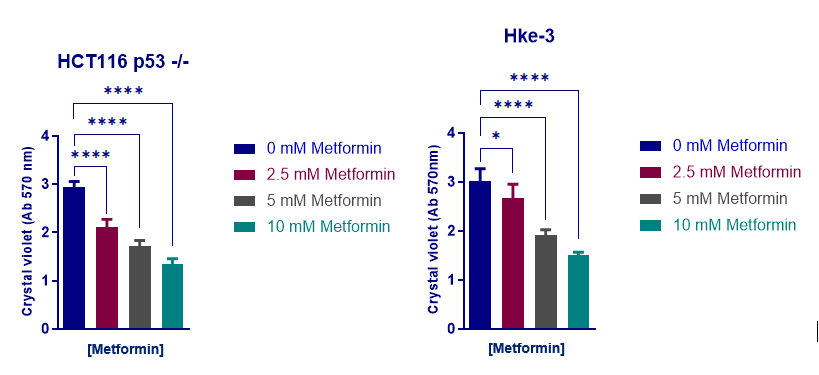

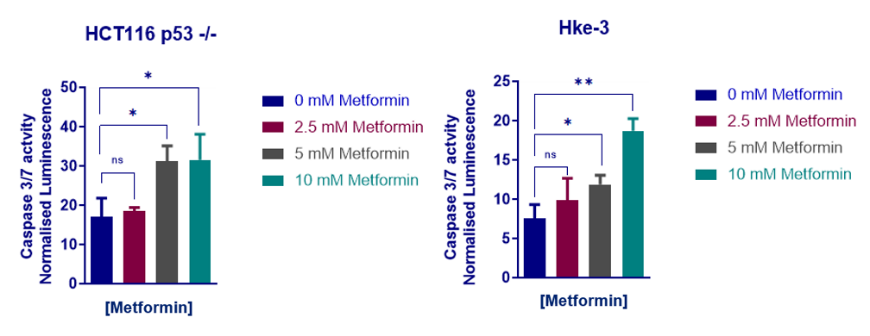


Supplementary Figure 1. Viability and apoptosis of HCT116, HCT116 TP53 null and Hke-3 (KRAS complemented) cells after 72 h of 2.5 mM, 5 mM and 10 mM metformin treatment.

(a) Crystal violet absorbance measurements in CRC cells treated with increasing doses of metformin, compared with cells in control medium. (b) Caspase 3/7 activity-related luminescence in CRC cells treated with increasing doses of metformin, compared with cells in control medium. Results are expressed as mean ± SD of at least 3 replicates and statistical significance is indicated with asterisks (* P ≤0.05, ** P ≤ 0.01, *** P ≤ 0.001, **** P ≤ 0.0001).

**(a) (b)**

Supplementary Figure 2. Pairwise correlation between the two independent replicates of the primary screen.

Results are based on quantification of normalized cell count in metformin (a) or vehicle treated (b) HCT116 cells 24 hours post-transfection with the library of miRNA mimics and are shown as cell count compared to the control siRNA (siOTP). Spearman rank correlation coefficients are shown.

**(a) (b)**
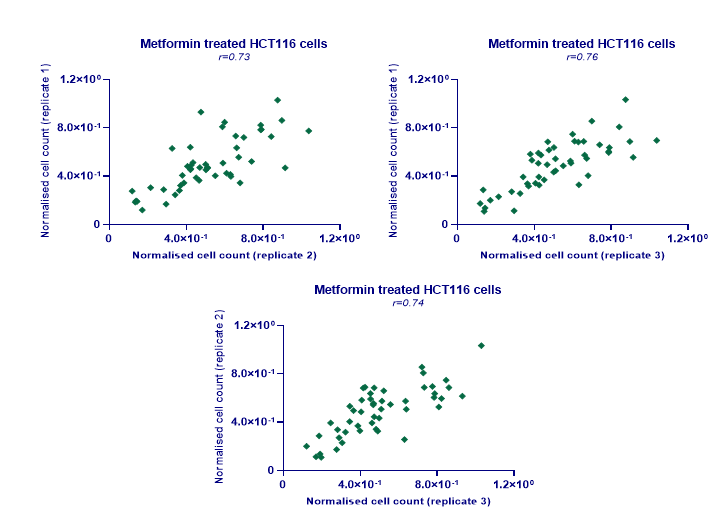

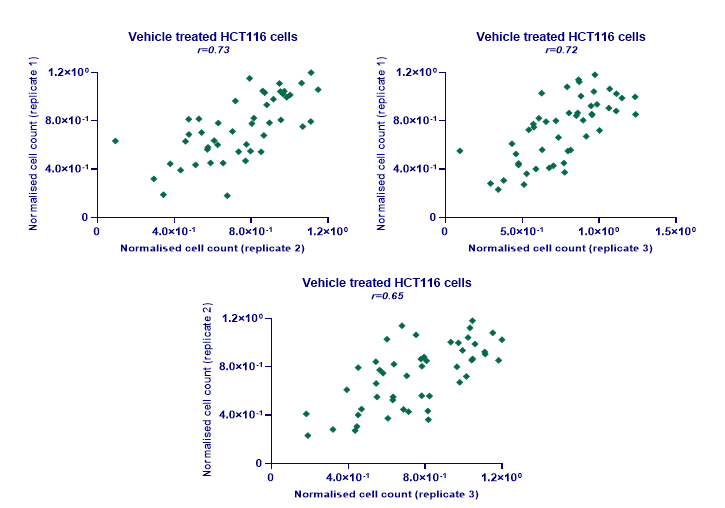


Supplementary Figure 3. Pairwise correlation between the three independent replicates of the secondary functional screen.

Results are based on quantification of normalized cell count in metformin (a) or vehicle treated (b) HCT116 cells 24 hours post-transfection with the library of miRNA mimics and are shown as cell count compared to the control siRNA (siOTP). Spearman rank correlation coefficients are shown.


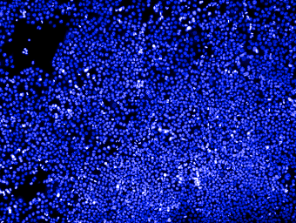

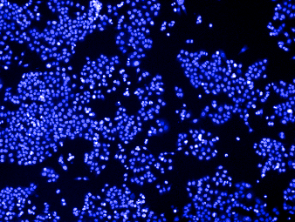


**NC**


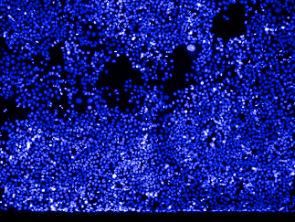

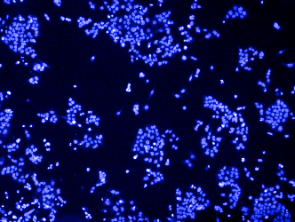


**miR-1181**


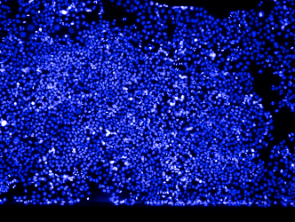

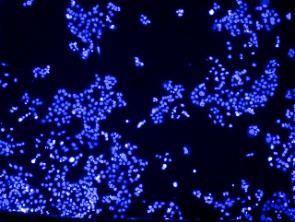


**miR-3687**


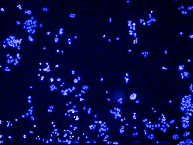

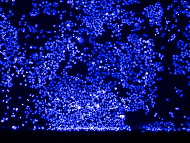


**miR-376b-5p**


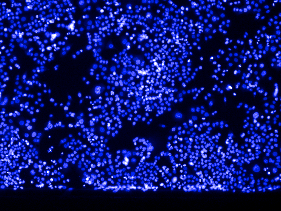

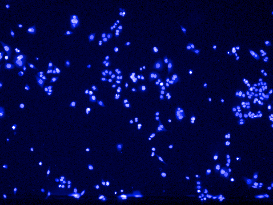


**miR-676-3p**


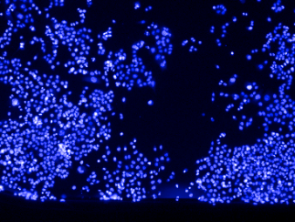

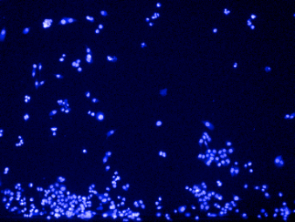


**miR-548v**


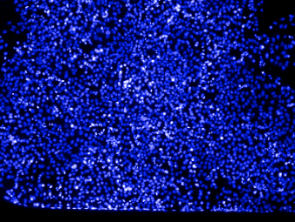

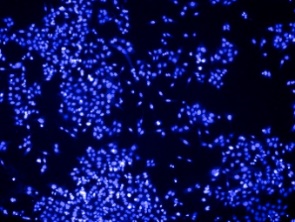


**miR-99a-3p**


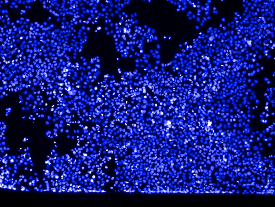

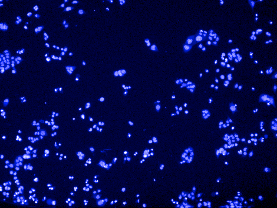


**miR-3187-3p**


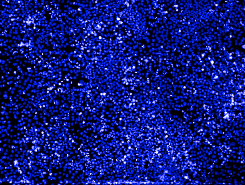

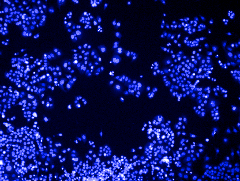


**miR-18b-5p**


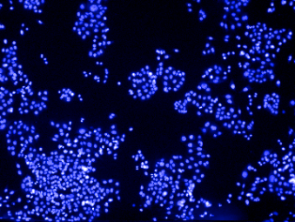

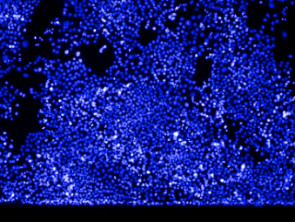


**miR-718**


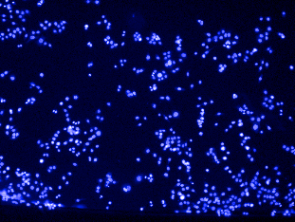

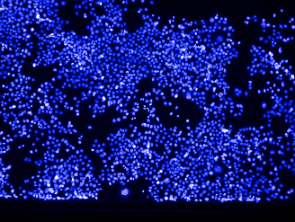


**miR-655-5p**


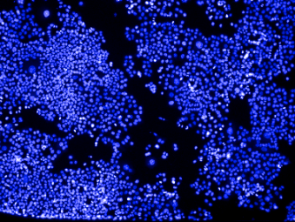

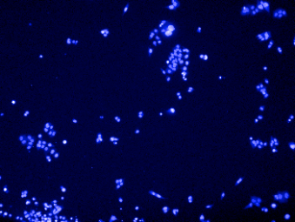


**miR-145-3p**

**Metformin 0 mM 2.5 mM 0mM 2.5 mM**

Supplementary Figure 4. Viability of HCT116 cells transfected with miRNA mimics having strong synergetic effect with 2.5 mM metformin treatment.

Representative images of HCT116 cells transfected with miRNA mimics, treated with 2.5 mM metformin and compared with 0 mM metformin treatment. Hoechst 33352 staining and image analysis shows viable cells with nucleus shown in blue.


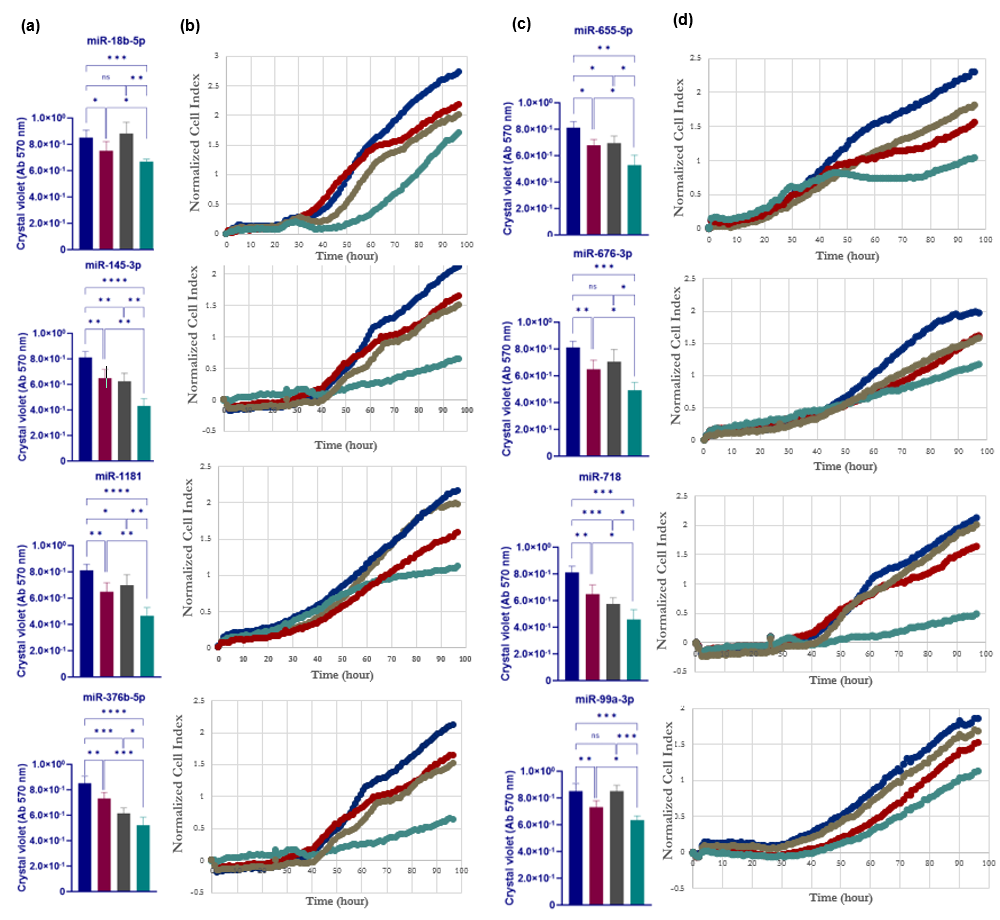


Supplementary Figure 5. Proliferation of HCT116 96 h post-miRNA mimic transfection and metformin treatment.

(a, c) Viability measurements at 96 h using crystal violet assays in HCT116 cells. Cells were transfected with miRNA mimics or NC and treated with 2.5 mM metformin or control medium 24 h post-transfection for 72 h. Results are expressed as mean ± SD of at least 3 culture replicates and the statistical significance is indicated with asterisk (ns P > 0.05, * P ≤0.05, ** P ≤ 0.01, *** P ≤ 0.001, **** P ≤ 0.0001). (b, d) xCELLigence real-time proliferation graphs demonstrating differences in HCT116 growth kinetics following NC or miRNA mimic transfection in control medium or 2.5 mM metformin. The average of two replicates is shown. The labels for growth curves in (a) and (c) correspond to those of the graphs in (b) and (d), respectively. Blue, red, grey and green bars and lines represent negative control, 2.5 mM metformin, miRNA mimic and miRNA mimic + 2.5 mM metformin, respectively.


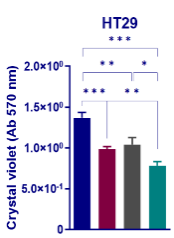

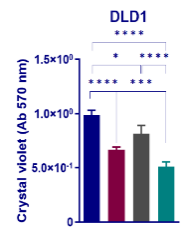

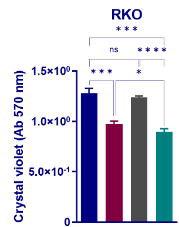

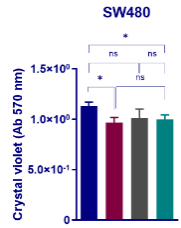

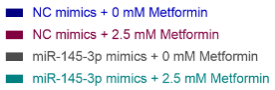

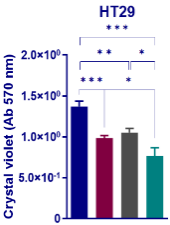

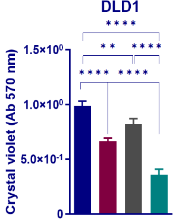

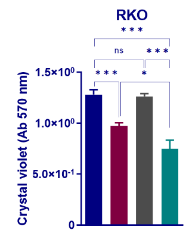

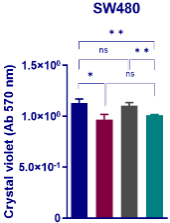

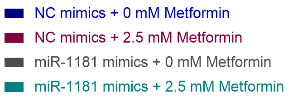

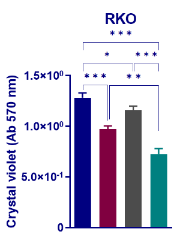

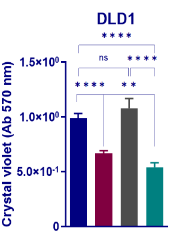

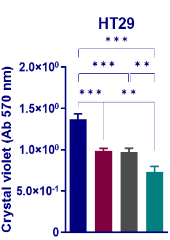

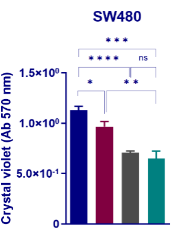

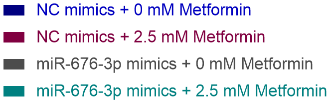

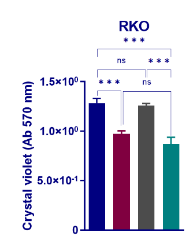

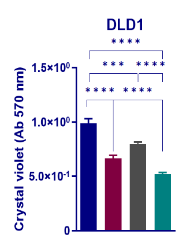

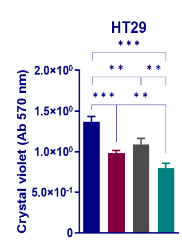

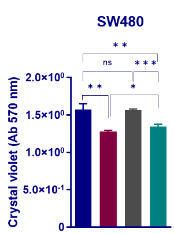

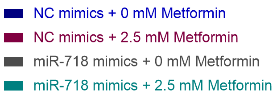

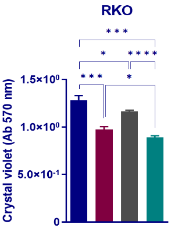

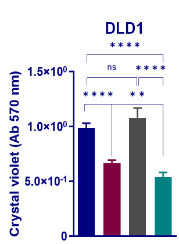

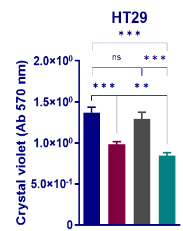

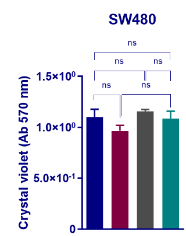

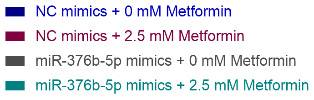

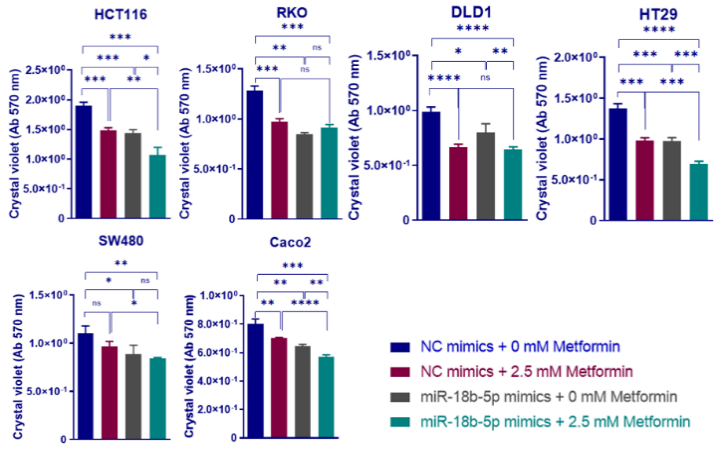

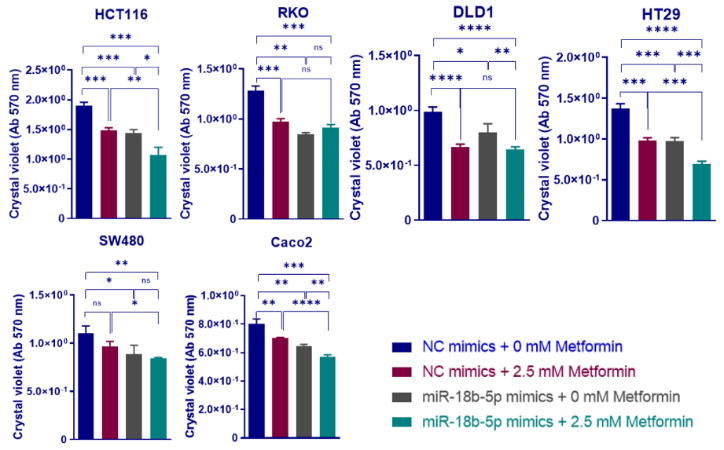

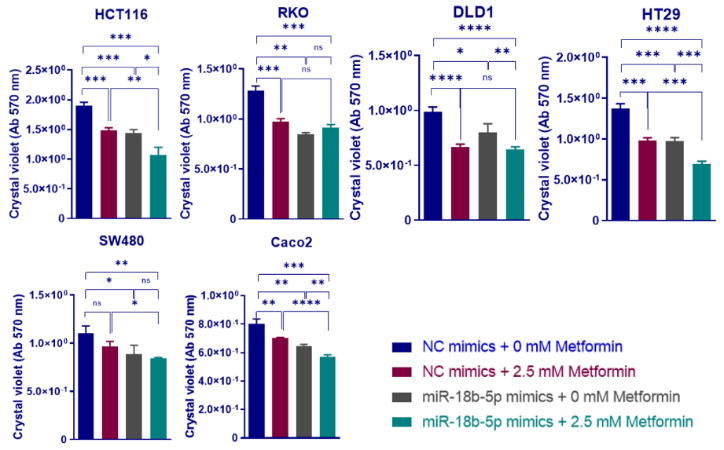

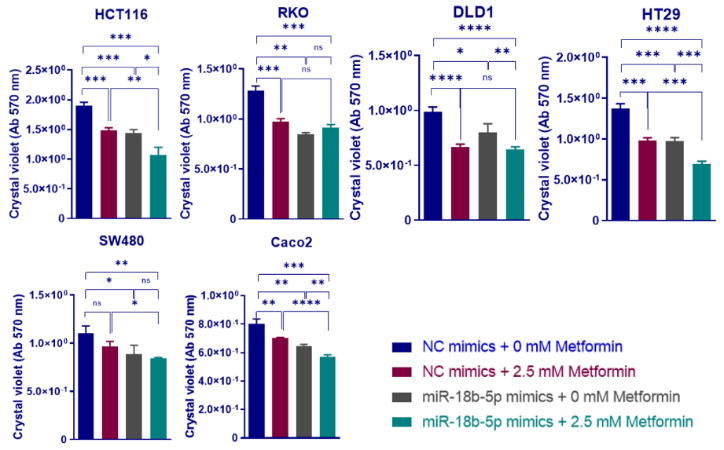


**(a)**

**(b)**

**(c)**

**(d)**

**(e)**

**(f)**

Supplementary Figure 6. Survival of different CRC cell lines after transfection with sensitizing miRNA mimic and treatment with metformin or control medium for 96 hours.

Viability measurements, by crystal violet assay, showing the different effects of (a) miR-18b-5p, (b) miR-145-3p, (c) miR-1181, (d) miR-376b-5p, (e) miR-676-3p and (f) miR-718 on proliferation, in the control medium and 2.5 mM metformin-treated RKO, DLD1, HT29 and SW480 cells. Results are expressed as mean ± SD of 3 technical replicates, statistical significance is indicated with asterisks (ns P > 0.05, * P ≤ 0.05, ** P ≤ 0.01, *** P ≤ 0.001 and **** P ≤ 0.0001).


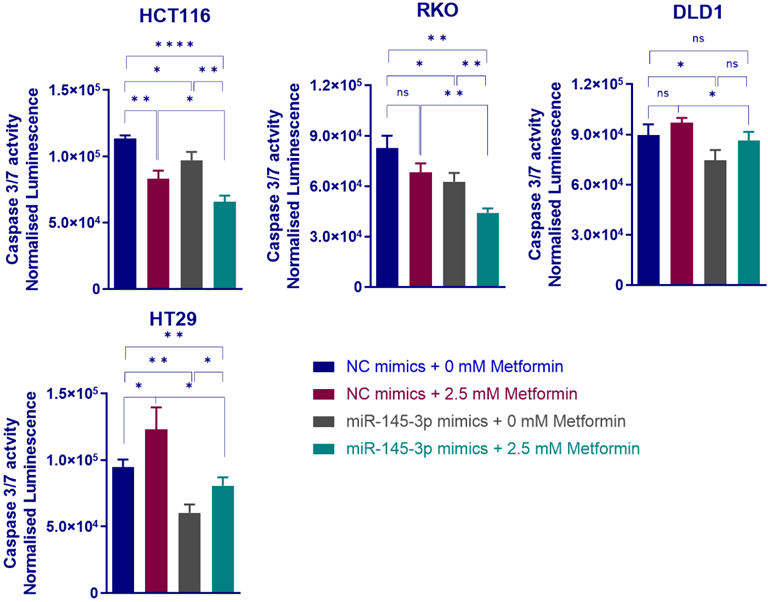

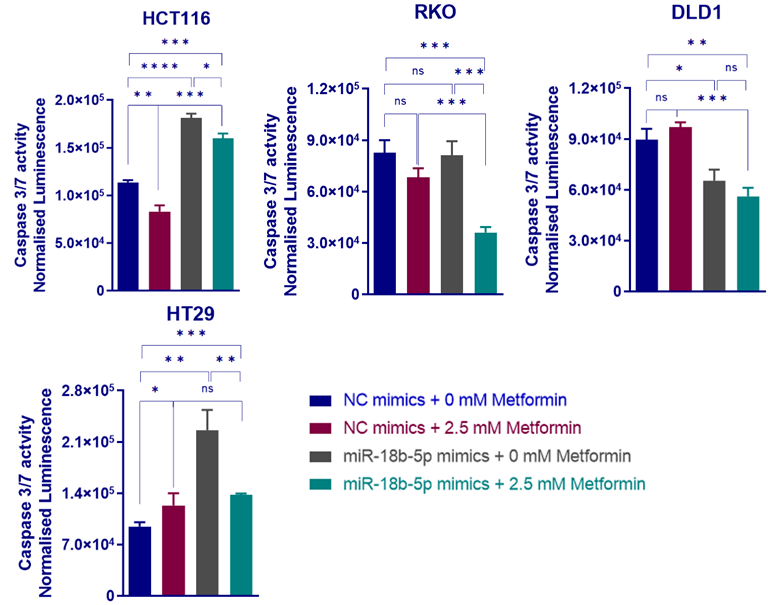

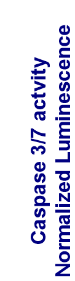

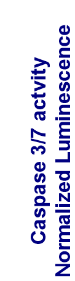

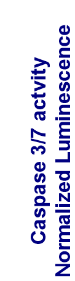

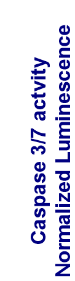

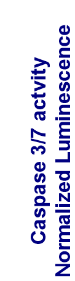

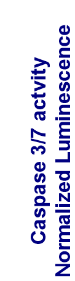

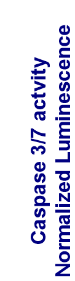

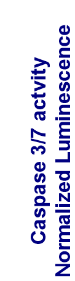

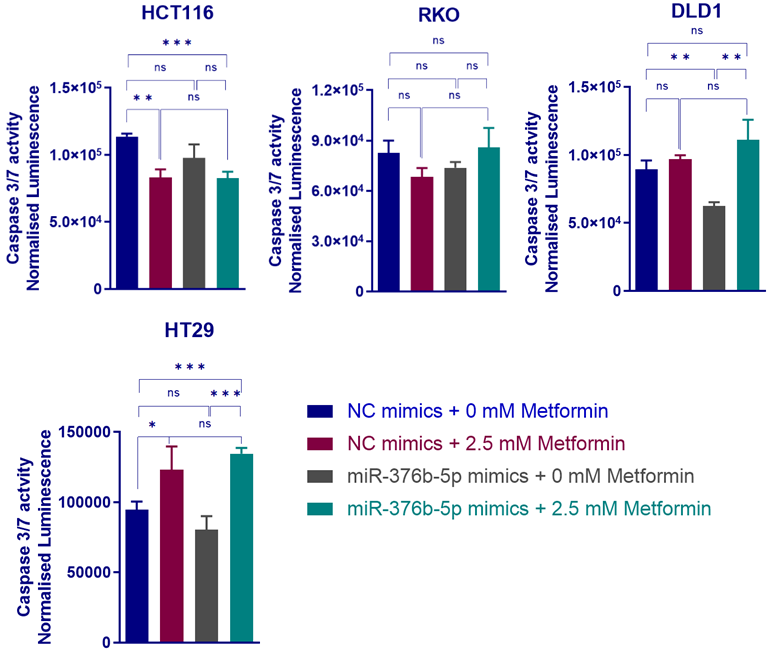

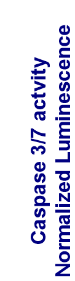

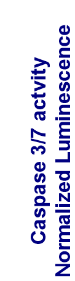

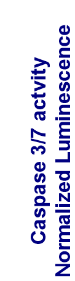

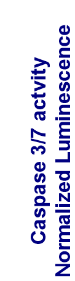

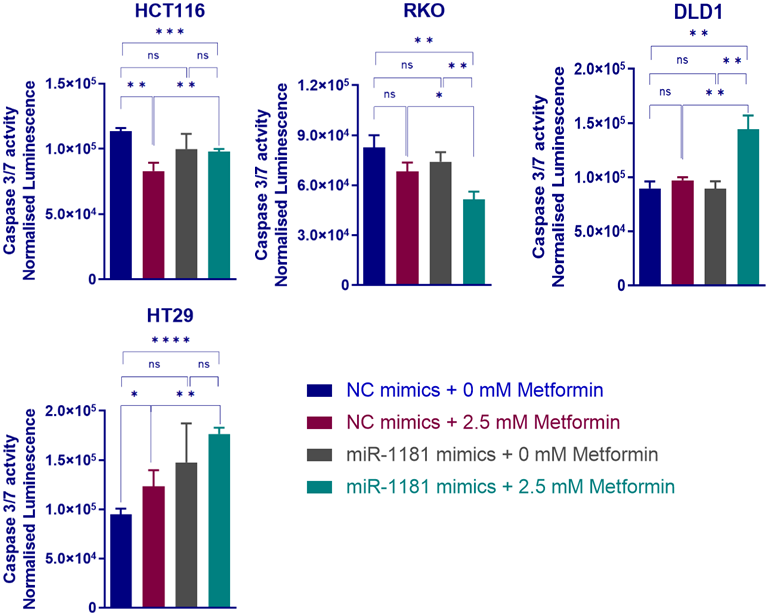

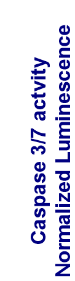

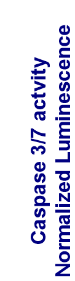

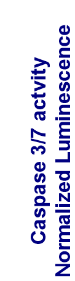

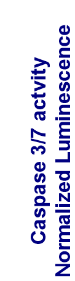

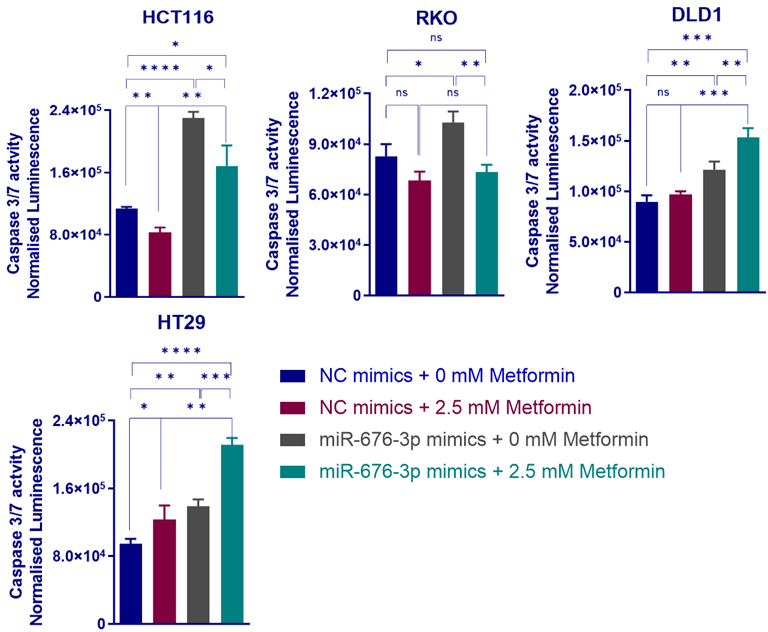

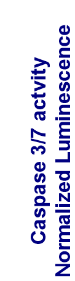

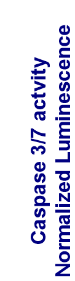

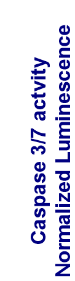

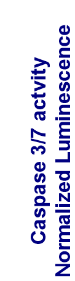

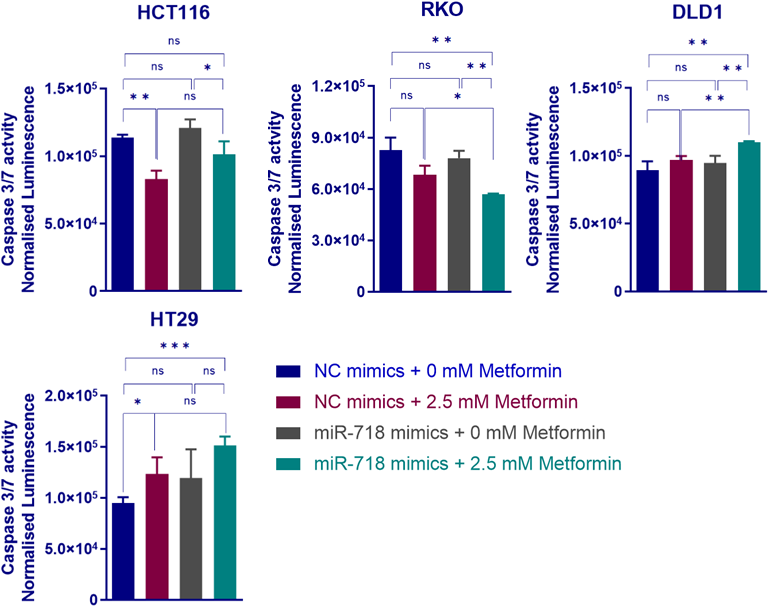

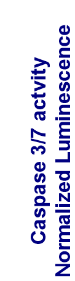

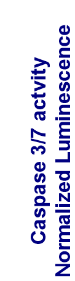

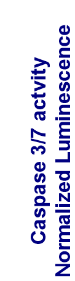

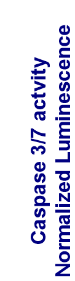


**(a)**

**(b)**

**(c)**

**(d)**

**(e)**

**(f)**

Supplementary Figure 7. Apoptosis of CRC cell lines after transfection with sensitizing miRNAs and treatment with metformin or control medium for 96 hours.

Caspase 3/7 activity luminescent signal measurements in HCT116, DLD1, RKO, and HT29 cells transfected with (a) miR-18b-5p, (b) miR-145-3p, (c) miR-1181, (d) miR-376b-5p, (e) miR-676-3p and (f) miR-718 mimics and treated with 2.5 mM metformin for 72 hours, compared with cells in control medium. Results are expressed as mean ± SD of 3 replicates and the statistical significance is indicated with asterisks (ns P > 0.05, * P ≤0.05, ** P ≤ 0.01, *** P ≤ 0.001).


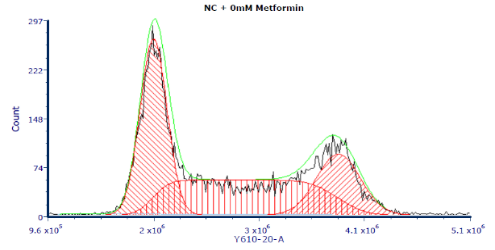

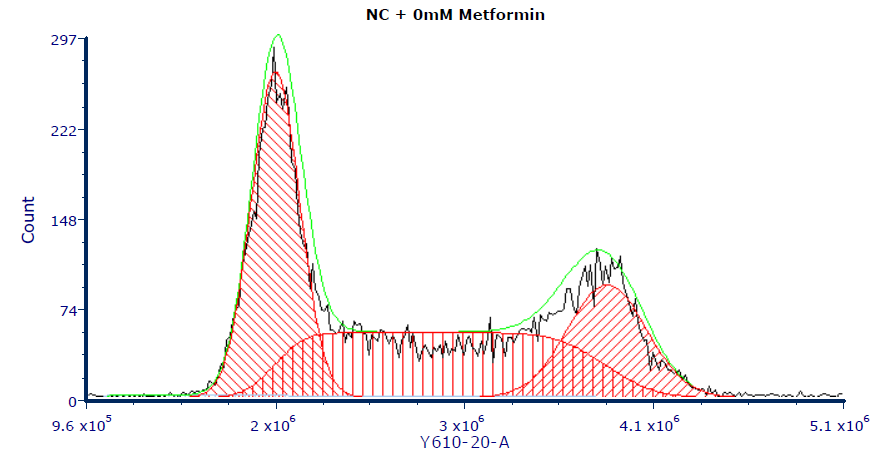

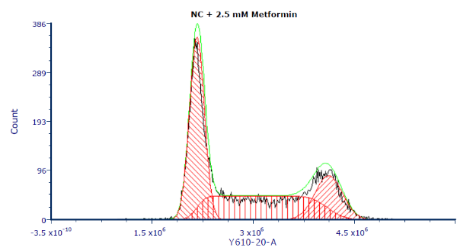

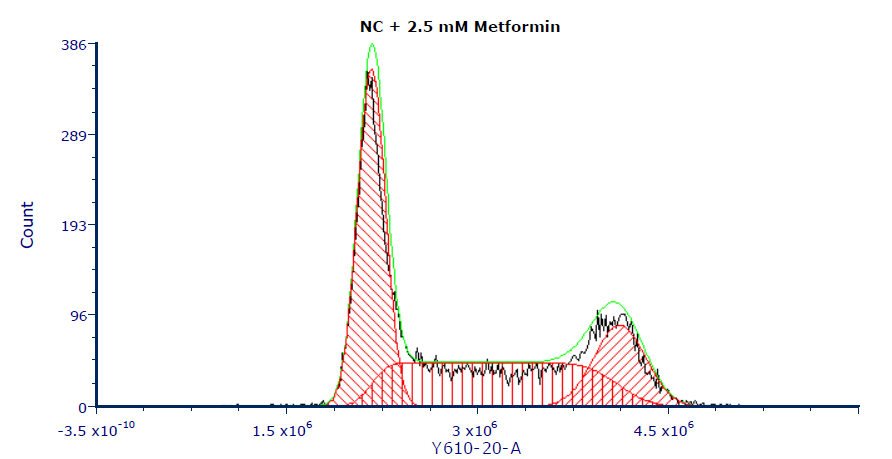

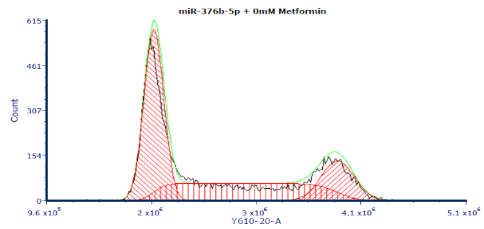


**(a)**

**(b)**

Supplementary Figure 8. Cell cycle distribution of HCT116 cells after transfection with sensitizing miRNAs and treatment with metformin or control medium for 96 hours.

(a) Representative results showing the distribution of the cells in G0/G1, S, or G2/M phase in HCT116 cells transfected with miR-18b-5p, miR-145-3p, miR-376b-5p, miR-676-3p or miR-718 mimics and treated with 2.5 mM metformin for 72 hours.(b) Example image of gating starategy for flow cytometry analyses.
